# Intestinal lysozyme liberates Nod1 ligands from microbes to direct insulin trafficking in pancreatic beta cells

**DOI:** 10.1038/s41422-019-0190-3

**Published:** 2019-06-14

**Authors:** Qin Zhang, Ying Pan, Benhua Zeng, Xiaojiao Zheng, Haifang Wang, Xueying Shen, Hui Li, Qian Jiang, Jiaxu Zhao, Zhuo-Xian Meng, Pingping Li, Zhengjun Chen, Hong Wei, Zhihua Liu

**Affiliations:** 1 Key Laboratory of Infection and Immunity, Institute of Biophysics, Chinese Academy of Sciences, Beijing, 100101 China; 2 Department of Laboratory Animal Science, College of Basic Medical Sciences, Third Military Medical University, Chongqing, 400038 China; 3 University of Chinese Academy of Sciences, Beijing, 100049 China; 4 The State Key Laboratory of Bioactive Substance and Function of Natural Medicines, Institute of Materia Medica, Chinese Academy of Medical Sciences & Peking Union Medical College, Beijing, 100050 China; 5 State Key Laboratory of Cell Biology, Shanghai Institute of Biochemistry and Cell Biology, Chinese Academy of Sciences, 320 Yueyang Rd, Shanghai, 200031 China; 6 Center for Excellence in Molecular Cell Science, Chinese Academy of Sciences, 320 Yueyang Rd, Shanghai, 200031 China; 7 Department of Pathology and Pathophysiology, School of Medicine, Zhejiang University, Hangzhou, Zhejiang 310058 China; 8 ShanghaiTech Univ, Sch Life Sci & Technol, 100 Haike Rd, Shanghai, 201210 China; 9 Precision Medicine Institute, The First Affiliated Hospital, Sun Yat-sen University, Guangzhou, Guangdong 510080 China; 10 Center for Excellence in Biomacromolecules, Chinese Academy of Sciences, Beijing, 100101 China

**Keywords:** Protein transport, Membrane trafficking

## Abstract

Long-range communication between intestinal symbiotic bacteria and extra-intestinal organs can occur through circulating bacterial signal molecules, through neural circuits, or through cytokines or hormones from host cells. Here we report that Nod1 ligands derived from intestinal bacteria act as signal molecules and directly modulate insulin trafficking in pancreatic beta cells. The cytosolic peptidoglycan receptor Nod1 and its downstream adapter Rip2 are required for insulin trafficking in beta cells in a cell-autonomous manner. Mechanistically, upon recognizing cognate ligands, Nod1 and Rip2 localize to insulin vesicles, recruiting Rab1a to direct insulin trafficking through the cytoplasm. Importantly, intestinal lysozyme liberates Nod1 ligands into the circulation, thus enabling long-range communication between intestinal microbes and islets. The intestine-islet crosstalk bridged by Nod1 ligands modulates host glucose tolerance. Our study defines a new type of inter-organ communication based on circulating bacterial signal molecules, which has broad implications for understanding the mutualistic relationship between microbes and host.

## Introduction

Commensal microbes play a vital role in modulating host metabolism.^1–3^ It is of critical importance to understand how the microbiota regulates host metabolism. Besides contributing nutrients by helping to digest otherwise indigestible dietary components and biosynthesizing essential metabolites, microbes are important in modulating host metabolism by communicating with the intestine or distant extra-intestinal organs. Long-distance communication between microbes and host organs can be achieved through direct communication by microbial signal molecules in the circulation system, indirect signaling through nerves, or through cytokines or hormones from host cells.^4^ The signal molecules can be structural constituents of the microbes, their secreted proteins or their metabolites.^4^ Many of the mechanisms that underlie the long-range crosstalk between intestinal microbiota and host organs still await discovery.

Colonization by intestinal microbes exerts complex influences on host metabolism. Intestinal colonization by bacteria is associated with enhanced energy harvesting, increased body fat content, and reduced insulin sensitivity.^1,3,5^ For instance, colonization of adult germ-free (GF) C57BL/6 mice with a normal microbiota produces relative insulin resistance within 14 days despite reduced food intake.^1^ Bacterial ligands modulate insulin sensitivity.^4^ Indeed, Nod1 ligands promote insulin resistance, especially in the murine model of diet-induced obesity (DIO), a phenomenon that has been attributed to the activation of innate immunity.^6–9^ However, whether the bacterial Nod1 ligands have more complex roles in metabolism besides promoting inflammation and insulin resistance is unclear.

To achieve glucose homeostasis, pancreatic beta cells integrate a multitude of metabolic, hormonal and neural cues to determine the extent of glucose-dependent insulin release.^10^ It has been discovered that pancreatic beta cells also integrate microbial signals to modulate insulin output. So far, two independent examples have been discovered. Acetate, a bacterial metabolite, stimulates the vagus nerve to promote insulin secretion from pancreatic beta cells.^11^ In zebra fish, a secreted protein from *Aeromonas* modulates beta cell expansion during early larval development through unknown mechanisms.^12^ Currently, it is unclear whether beta cells are able to directly sense microbial signal molecules to modulate insulin output.

Insulin biogenesis starts in the rough endoplasmic reticulum (ER) where preproinsulin is synthesized and converted to proinsulin. Proinsulin is transported to the Golgi and sorted into immature dense core vesicles (DCVs), which bud off from the trans-Golgi network (TGN). DCVs undergo an as yet poorly defined maturation process that involves homotypic vesicle fusion, acidification, conversion of proinsulin to insulin, and the removal of some soluble and transmembrane cargos. As the conversion process occurs, DCVs travel through the cytosol, usually along the microtubules, until they come into close proximity with the plasma membrane, where they usually move along microfilaments and eventually fuse with the plasma membrane in a glucose-dependent manner. Thus, the insulin biogenesis process includes insulin synthesis, insulin granule sorting, maturation, distribution, signaling pathway and exocytosis.^13,14^ Currently, the intermediate part of this process, including insulin granule sorting, maturation and distribution, remains poorly defined. The individual steps are deeply intertwined and are sometimes generally termed as insulin intracellular trafficking.

In this study, we probe for the effect of microbial colonization on insulin trafficking in pancreatic beta cells. We find that the presence of microbiota modulates insulin distribution in islet beta cells. Nod1 expressed in beta cells senses the intestine-derived Nod1 ligands, translocates to insulin granules, and recruits downstream Rip2 and Rab1a to promote insulin granule transport. Interestingly, intestinal lysozyme from Paneth cells is required for releasing Nod1 ligands from commensal bacteria. Microbe-sensing through Nod1 is required for efficient glucose-stimulated insulin secretion (GSIS). Finally, specific deficiency of Nod1 in beta cells impairs glucose tolerance. Collectively, our study identifies a new intestine-islet axis important for host glucose tolerance, in which beta cells directly sense microbial Nod1 ligands released from commensal bacteria by intestinal lysozyme.

## Results

### Intestinal microbes affect insulin distribution in pancreatic beta cells in a cell-autonomous manner

**Fig. 1:**
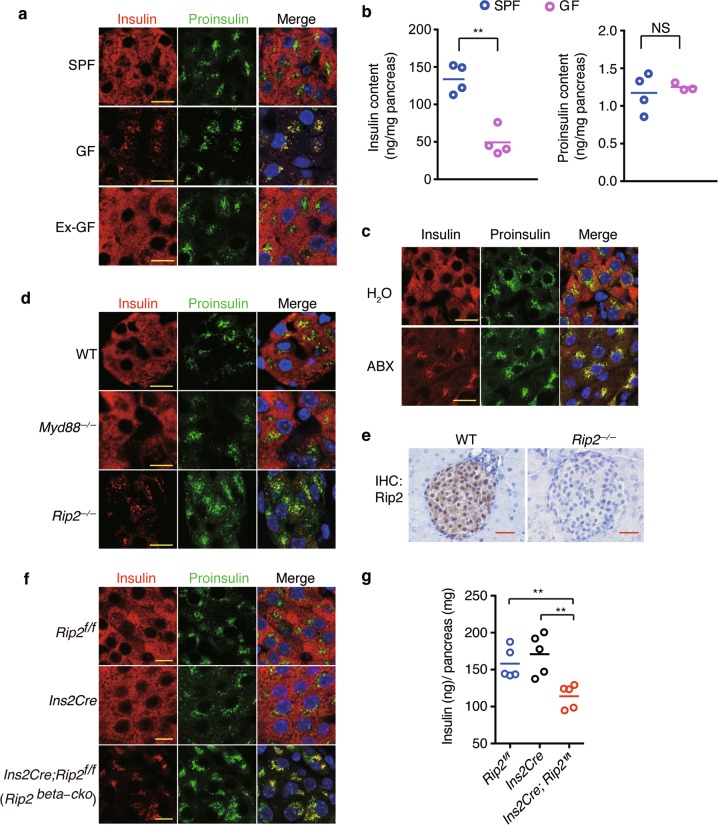
Beta cells sense microbes to direct insulin distribution in a cell-autonomous manner. **a** Immunostaining and confocal imaging of insulin (red) and proinsulin (green) in paraffin sections of pancreata from SPF, GF, and ex-GF mice. **b** The amount of insulin and proinsulin in pancreatic tissues from SPF and GF mice. **c** Immunostaining and confocal imaging of insulin (red) and proinsulin (green) in paraffin sections of  pancreata from H_2_O (vehicle)- or antibiotic cocktail (ABX)-treated mice. **d** Immunostaining and confocal imaging of insulin and proinsulin in paraffin sections of pancreata from wild-type (WT), *myd88*^*−/−*^ and *Rip2*^*−/−*^ mice. **e** Immunohistochemical staining (IHC) of Rip2 in paraffin sections of pancreata from WT and *Rip2*^*−/−*^ mice. **f** Immunostaining and confocal imaging of insulin and proinsulin in paraffin sections of pancreata from *Rip2*^*f/f*^, *Ins2Cre*, and *Rip2*^*beta-cko*^ mice. **g** The amount of insulin in pancreatic tissues from *Rip2*^*f/f*^, *Ins2Cre*, and *Rip2*^*beta-cko*^ mice. Nuclei were counter-stained in blue (**a**, **c**, **d**–**f**). Scale bars, 10 μm in **a**, **c**, **d**, **f**, 50 μm in **e**. Each symbol represents an individual animal, and horizontal bars indicate median values (**b**, **g**). *P* values were calculated with a two-tailed Student’s *t-*test (**b**) and a one-way ANOVA (**g**). **P < 0.01, NS, *P* > 0.05. Data (**a**–**f**) are representative of three independent experiments

To understand whether insulin trafficking in beta cells is affected by intestinal microbes, we examined the cellular distribution of insulin and proinsulin in islets from conventionally raised specific pathogen-free (SPF) mice, germ-free (GF) mice and colonized GF (ex-GF) mice, by immunofluorescence staining and confocal imaging. In beta cells from SPF mice, insulin and proinsulin staining was clearly segregated, with insulin^+^ mature DCVs dispersed ubiquitously throughout the cytoplasm and proinsulin^+^ immature DCVs restricted to the perinuclear region (Fig. 1a). This segregated distribution pattern of proinsulin^+^ vesicles and insulin^+^ vesicles is consistent with other reports,^15,16^ and likely represents the ordered maturation process in beta cells under physiological conditions.

However, the segregated distribution pattern of insulin and proinsulin was disturbed in GF and antibiotic cocktail (ABX)-treated mice. Insulin colocalized with proinsulin in the perinuclear region in beta cells in GF islets (Fig. 1a). Also, the overall insulin staining in beta cells was weaker in GF mice than in SPF mice, and quantitative analysis of protein extracts confirmed that the pancreata in GF mice contained significantly less insulin than those in SPF mice (Fig. 1b). We chose to quantify the amounts of insulin and proinsulin from protein extracts  of  whole pancreatic tissues, because the distribution of islets can vary significantly throughout the pancreas.^17,18^ The amount of proinsulin in pancreata in GF mice was not significantly different from those in SPF mice (Fig. 1b). In ex-GF beta cells, the segregated distribution of insulin and proinsulin was restored (Fig. 1a). Conversely, ABX treatment led to colocalization of insulin and proinsulin in islet beta cells (Fig. 1c). Thus, the presence of commensal bacteria affects insulin distribution in beta cells.

We wondered whether certain pattern recognition receptors (PPRs) might be directly or indirectly involved in sensing microbes to affect the intracellular distribution of insulin vesicles in beta cells. We first examined the intracellular distribution of insulin in *myd88*^*−/−*^ or *Rip2*^*−/−*^ mice. Myd88 is the major adapter downstream of membrane-associated toll-like receptors (TLRs), while Rip2 is the key adapter downstream of two cytosolic peptidoglycan receptors, Nod1 and Nod2.^19–21^ The intracellular distribution of insulin and proinsulin in *Myd88*^*−/−*^ mice was comparable with that in wild-type (WT) mice (Fig. 1d). In comparison, insulin colocalized with proinsulin in the perinuclear region in *Rip2*^*−/−*^ mice (Fig. 1d), similar to the insulin staining pattern observed in GF mice. Our immunohistochemical (IHC) staining showed that Rip2 was highly expressed throughout the islet (Fig. 1e), suggesting the possibility that Rip2 is required autonomously in beta cells for proper insulin distribution. To test this possibility, we generated *Ins2Cre*; *Rip2*^*f/f*^ mice (with loxP-flanked Rip2 alleles (*Rip2*^*f/f*^) deleted by Cre recombinase expressed in pancreatic beta cells), which specifically lack Rip2 (Supplementary Information, Fig. S1a–c). These mice are hereafter designated as *Rip2*^*beta-cko*^. Specific loss of Rip2 in beta cells led to the co-localization of insulin and proinsulin in the perinuclear region (Fig. 1f) and a reduction of insulin content in pancreatic tissues in *Rip2*^*beta-cko*^ mice (Fig. 1g). The reduction of insulin content in pancreatic tissues in *Rip2*^*beta-cko*^ mice was not due to reduced expression of insulin, because the insulin RNA (transcripts from *Ins1* and *Ins2*) level from *Rip2*^*beta-cko*^ mice was not different from that from the control mice (Supplementary Information, Fig. S1d). Therefore, we conclude that Rip2 is required in a cell-autonomous manner in beta cells to direct insulin distribution.

### Nod1 ligands from intestinal bacteria direct insulin distribution in beta cells

**Fig. 2:**
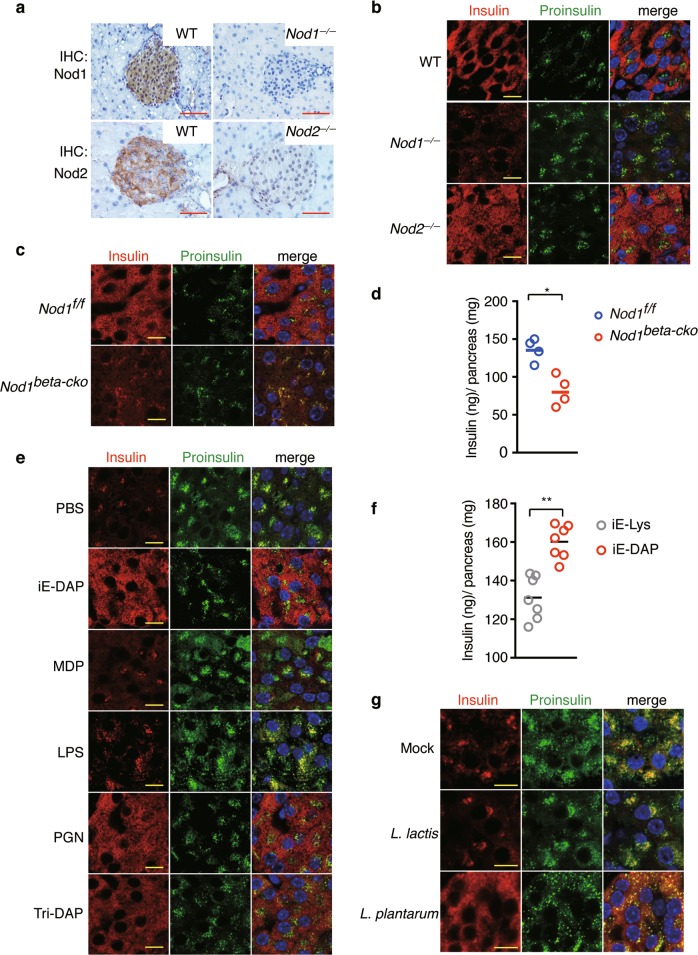
Nod1 ligands from intestinal flora direct insulin distribution in beta cells. **a** IHC staining of Nod1 or Nod2 in paraffin sections of pancreata from WT, *Nod1*^*−/−*^ and *Nod2*^*−/−*^ mice. **b**, **c** Immunostaining and confocal imaging of insulin and proinsulin in paraffin sections of pancreata from WT, *Nod1*^*−/−*^ and *Nod2*^*−/−*^ mice (**b**), and from *Nod1*^*f/f*^ and *Nod1*^*beta-cko*^ mice (**c**). **d** The amount of insulin in pancreatic tissues from *Nod1*^*f/f*^, and *Nod1*^*beta-cko*^ mice. **e** Immunostaining and confocal imaging of insulin and proinsulin in paraffin sections of pancreata from GF mice treated with PBS as vehicle, iE-DAP, MDP, LPS, PGN (peptidoglycan from *Bacillus subtilis*), or Tri-DAP. **f** The amount of insulin in pancreatic tissues from GF mice orally administrated with either iE-Lys or iE-DAP. **g** Immunostaining and confocal imaging of insulin and proinsulin in paraffin sections of pancreata from mice mono-colonized with *L. lactis* strain NZ9000 or *L. plantarum* strain NY, or mock-treated (PBS). Nuclei were counter-stained in blue (**a**–**c**, **e**, **g**). Scale bars, 50 μm in **a**, 10 μm in **b**, **c**, **e**, **g**. *P* values were calculated with a two-tailed Student’s *t-*test (**d**, **f**). *P < 0.05, **P < 0.01. Data are representative of three independent experiments

Rip2 is the key adapter downstream of Nod1 and Nod2. The expression of Nod1 and Nod2 in islets was detected by IHC (Fig. 2a). We examined the insulin distribution pattern in islets from *Nod1*^*−/−*^ and *Nod2*^*−/−*^ mice with confocal imaging (Fig. 2b). Insulin colocalized with proinsulin in the perinuclear region in *Nod1*^*−/−*^ mice, while the distribution of insulin in *Nod2*^*−/−*^ mice was similar with that in WT mice (Fig. 2b). This indicates that Nod1 is the receptor responsible for the microbe-mediated insulin distribution. Since Rip2 is required cell-autonomously in beta cells for insulin trafficking, we speculated that Nod1 was also required for insulin distribution in beta cells in a cell autonomous manner. To test this, we generated *Ins2Cre*; *Nod1*^*f/f*^ mice (with loxP-flanked Nod1 alleles (*Nod1*^*f/f*^) deleted by Cre recombinase expressed in pancreatic beta cells), designated as *Nod1*^*beta-cko*^ hereafter (Supplementary Information, Fig. S2a–c). Specific loss of Nod1 in islet beta cells led to perinuclear distribution of insulin vesicles (Fig. 2c) and a reduction of insulin content in pancreatic tissues from *Nod1*^*beta-cko*^ mice (Fig. 2d), confirming our hypothesis that Nod1 is required cell-autonomously in beta cells to sense microbial signals.

To understand whether the intracellular insulin distribution depends on intestine-derived bacterial ligands, we treated GF mice with various bacterial ligands by oral gavage. Supplementation with γ-D-Glu-meso-diaminopimelic acid (iE-DAP), L-Ala-γ-D-Glu-meso-diaminopimelic acid (Tri-DAP), or peptidoglycan (PGN) from *Bacillus subtilis*, induced the dispersed distribution of insulin in GF mice, but other bacterial ligands, such as muramyl dipeptide (MDP) or lipopolysaccharide (LPS) had no effect (Fig. 2e). Both iE-DAP and Tri-DAP are cognate Nod1 ligands, and PGN from *B. subtilis* includes the Nod1 ligand motif.^22^ Consistently, supplementing GF mice with iE-DAP, but not with the inactive analog iE-Lys, increased insulin content in pancreatic tissues (Fig. 2f). Thus, Nod1 ligands in the purified form are sufficient to direct insulin distribution. We next sought to determine whether insulin distribution requires the presence of a complex bacterial community, such as intestinal microbiota, or a single species of bacterium containing a Nod1 ligand. We inoculated GF mice with a single species of *Lactobacillus plantarum*, which has a DAP-type PGN. As the control, we also inoculated GF mice with a single species of *Lactococcus lactis*, which has a Lys-type PGN. We found that mono-colonization with *L. plantarum* induced the dispersed distribution of insulin, but mono-colonization with *L. lactis* did not (Fig. 2g). The ability of *L. plantarum* to restore insulin distribution was not strain-dependent, because mono-colonization with another strain of *L. plantarum* also restored the cytosolic distribution of insulin in beta cells (Supplementary Information, Fig. S2d). These results suggest that Nod1 ligands from intestinal bacteria are responsible for directing insulin distribution in beta cells, and such an effect does not rely on the presence of a complex intestinal microbiota.

### Nod1 and Rip2 localize to DCVs and modulate the cellular distribution of insulin vesicles

**Fig. 3:**
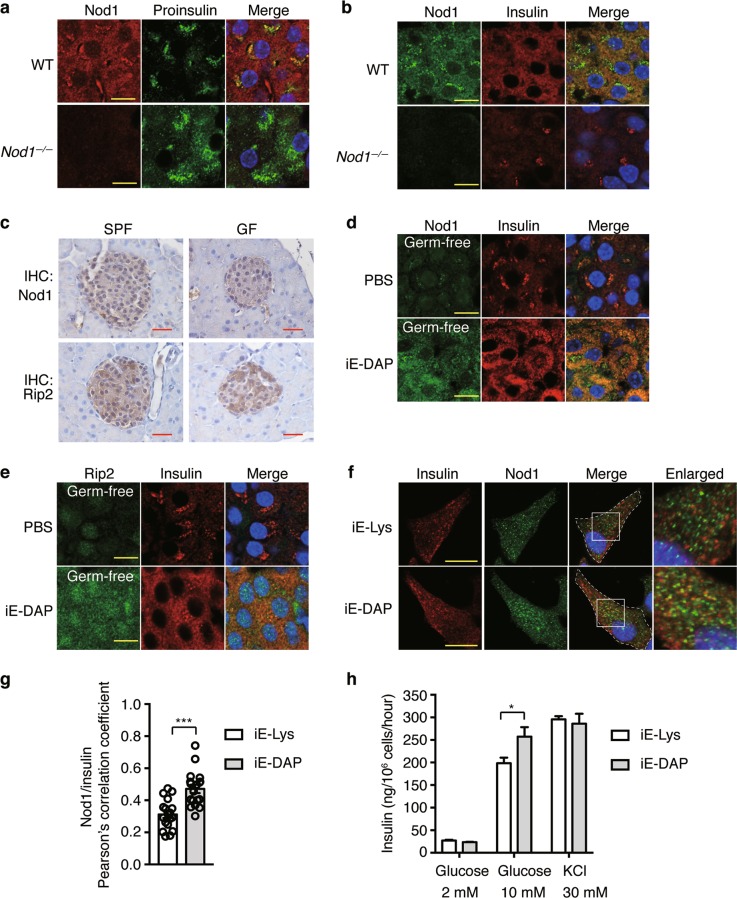
Nod1 ligands recruit Nod1 and Rip2 onto insulin vesicles in beta cells. **a, b** Immunostaining and confocal imaging of Nod1 and proinsulin (**a**) or insulin (**b**) in paraffin sections of pancreata from WT and *Nod1*^*−/−*^ mice. **c** IHC staining of Nod1 or Rip2 in paraffin sections of pancreata from SPF or GF mice. **d**, **e** Immunostaining and confocal imaging of Nod1 (**d**) or Rip2 (**e**) and insulin in paraffin sections of pancreata from PBS- or iE-DAP-treated GF mice. **f** Immunostaining and confocal imaging of insulin and Nod1 in INS-1 cells treated with iE- Lys or iE-DAP for 6 h. White dashed lines outline the cell boundaries. Boxed areas are enlarged on the right. **g** The quantification of colocalization between Nod1 and Insulin in **f**. Pearson’s correlation coefficients were calculated. For each experiment, 18 cells were analyzed. **h** The insulin amount in supernatants of INS-1 832/13 cells incubated in KRBH buffer with designated concentrations of glucose or KCl. Nuclei were counter-stained in blue (**a**–**f**). Scale bars, 10 μm (**a, b, d**–**f**), 50 μm (**c**). DIC, differential interference contrast. *P* values were calculated with a two-tailed Student’s *t-*test (**g**) or a one-way ANOVA followed by Tukey’s post hoc tests (**h**). *P *<* 0.05, ***P *<* 0.001. Data are representative of three independent experiments

To determine how Nod1 and Rip2 are required for insulin distribution in beta cells, we first determined the cellular localization of Nod1 and Rip2. Confocal imaging showed that Nod1 predominantly localized to immature proinsulin^+^ DCVs and mature insulin^+^ DCVs (Fig. 3a, b). Nod1 did not colocalize with calnexin, an endoplasmic reticulum resident protein, or GM 130, a Golgi-resident protein (Supplementary Information, Fig. S3a, b). Nod1 and Rip2 showed a high degree of co-localization in beta cells (Supplementary Information, Fig. S3c), indicating that Rip2 shares a similar distribution pattern with Nod1 on DCVs.

Nod1 and Nod2 are cytosolic PGN sensors; however, studies have found that ligand binding can trigger the translocation of Nod1 and Nod2 onto the membranes of endosomes or DCVs.^23–25^ We were prompted to examine whether DCV localization of Nod1 in beta cells is also ligand-dependent. First, IHC and Q-PCR showed comparable levels of Nod1 and Rip2 protein and mRNA in islets from SPF or GF mice (Fig. 3c; Supplementary Information, Fig. S3d). However, confocal imaging showed a greatly diminished Nod1 signal on DCVs (visualized by insulin staining) in GF mice, and iE-DAP supplementation restored Nod1 on DCVs (Fig. 3d). A similar phenomenon was observed for Rip2 (Fig. 3e). Thus, Nod1 and Rip2 localize to DCVs in a ligand-dependent manner in beta cells in vivo.

To determine whether Nod1 ligands directly promote Nod1 recruitment onto DCVs, we analyzed the effect of iE-DAP on INS-1 cells, a rat insulinoma cell line.^26^ INS-1 cells express both Nod1 and Rip2 (Supplementary Information, Fig. S3e). Immunofluorescence staining showed only a small fraction of insulin vesicles were positive for Nod1 staining in untreated INS-1 cells, and iE-DAP treatment significantly increased Nod1 staining on insulin vesicles (Fig. 3f, g). INS-1 832/13 cells, a subclone derived from INS-1 cells, potently secrete insulin in response to glucose stimulation.^27,28^ We performed glucose-induced insulin secretion (GSIS) assay using a method which optimized insulin secretion in INS-1 832/13 cells.^29^ In INS-1 832/13 cells, the treatment of iE-DAP increased GSIS compared to iE-Lys treatment (Fig. 3h). As comparison, treatment of iE-DAP did not alter insulin secretion upon KCl treatment (Fig. 3h). The increased secretion of insulin was independent of insulin mRNA transcription, because the mRNA level of insulin remained unchanged between iE-DAP and iE-Lys treatments (Supplementary Information, Fig. S3f). Taken together, intestine-derived Nod1 ligands recruit Nod1 and Rip2 onto DCVs in vivo and in vitro, which may affect the intracellular distribution of insulin vesicles.

### Nod1 and Rip2 recruit Rab1a onto DCVs to regulate insulin trafficking

Rabs, a family of small GTPases, are master regulators of intracellular membrane trafficking. Rab1, a *Drosophila* analog of Rab1a, controls insulin-like peptide trafficking in insulin-producing cells (IPCs) in *Drosophila melanogaster.*^30^ Inhibition of Rab1 leads to reduced distribution of an insulin-like peptide to the axonal projection in IPCs in *Drosophila.*^30^ Despite being known as a Golgi-resident GTPase, Rab1a also localizes to the membranes of lysosome-like organelles, such as insulin vesicles, melanosomes and early endosomes.^31–34^ Studies have shown that Rab1a regulates anterograde transport of melanosomes along the microtubules in melanocytes.^33,35^ Thus, we suspected that Rab1a might act downstream of Nod1 and Rip2 in regulating DCV transport.

**Fig. 4:**
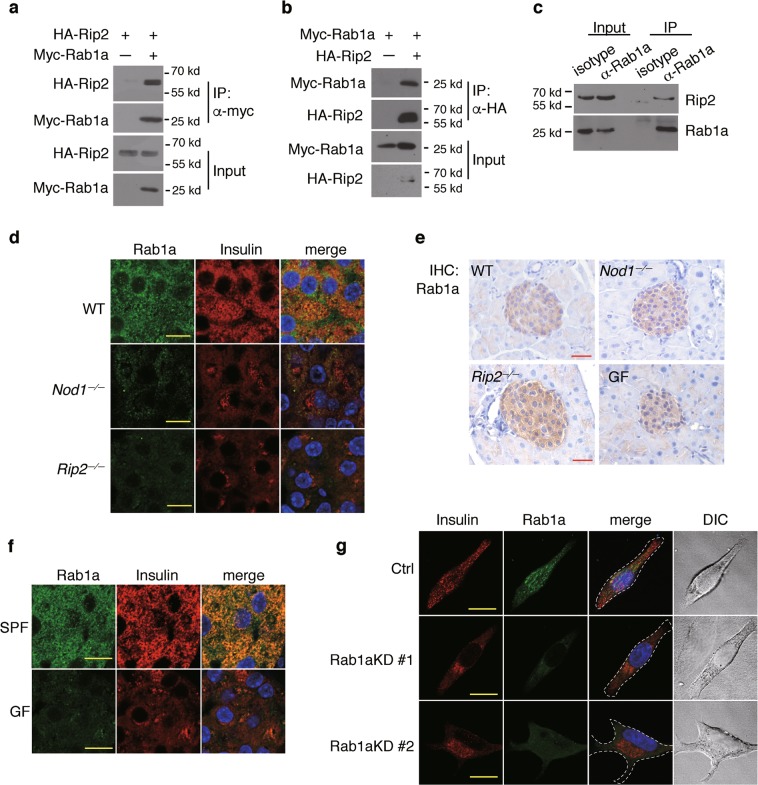
Nod1 and Rip2 recruit Rab1a onto DCVs to direct insulin vesicle transport. **a**, **b** Co-immunoprecipitation (IP) of Rip2 and Rab1a overexpressed in HEK293T cells. Tagged proteins were expressed in HEK293T cells, IP with anti-myc (**a**) or anti-HA (**b**) beads, and immunoblotted. **c** Co-IP of endogenous Rip2 and Rab1a in INS-1 cells. Rab1a was precipitated with an anti-Rab1a antibody. Rip2 and Rab1a were immunoblotted in input and IP fractions. **d** Immunostaining and confocal imaging of Rab1a and insulin in paraffin sections of pancreata from WT, *Nod1*^*−/−*^ and *Rip2*^*−/−*^ mice. **e** IHC staining of Nod1 or Rip2 in paraffin sections of pancreata from WT, *Nod1*^*−/−*^, *Rip2*^*−/−*^, and GF mice. **f** Immunostaining and confocal imaging of Rab1a and insulin in paraffin sections of pancreata from SPF and GF mice. **g** Immunostaining and confocal imaging of insulin and Rab1a in INS-1 cells with stable shRNA knockdown of Rab1a. Ctrl, scramble shRNA; Rab1aKD #1 or Rab1aKD #2, shRNAs against Rab1a. Nuclei were counter-stained in blue (**d**–**g**). Scale bars, 50 μm (**e**), 10 μm (**d**, **f**, **g**). Data are representative of three independent experiments

Using immunoprecipitation, we found that Rab1a interacted with Rip2 when these proteins were overexpressed in HEK293T cells (Fig. 4a, b). The interaction between endogenous Rab1a and Rip2 in INS-1 cells was confirmed by immunoprecipitation (Fig. 4c). Rab1a colocalized extensively with insulin in beta cells in WT mice (Fig. 4d), consistent with previous reports that Rab1a is found on insulin vesicles.^31,32^ By comparison, the recruitment of Rab1a to insulin^+^ DCVs was abolished in beta cells in *Nod1*^*−/−*^ or *Rip2*^*−/−*^ mice (Fig. 4d). This was not due to reduced expression of Rab1a, because comparable levels of Rab1a were detected in islets from WT, *Nod1*^*−/−*^ and *Rip2*^*−/−*^ mice (Fig. 4e; Supplementary Information, Fig. S4a, b). We next examined the DCV location of Rab1a in beta cells in GF mice. Beta cells in GF and SPF mice expressed similar levels of Rab1a (Fig. 4e), but the localization of Rab1a on insulin^+^ DCVs was greatly diminished in GF mice compared to SPF mice (Fig. 4f); this could be restored by iE-DAP supplementation (Supplementary Information, Fig. S4c). Therefore, the recruitment of Rab1a onto insulin vesicles depends on Nod1, Rip2 and microbial signals.

We wanted to test whether Rab1a is indeed involved in insulin intracellular trafficking. Rab1a is involved in ER-Golgi transport, and direct knockout of Rab1a may cause Golgi fragmentation.^36^ Stable knockdown of Rab1a using shRNA did not lead to Golgi abnormalities in INS-1 cells (Supplementary Information, Fig. S4d, e), consistent with previous reports in melanocytes.^33,35^ In INS-1 cells treated with scrambled shRNA, insulin vesicles were more dispersed in the cytosol whereas following Rab1a knockdown, insulin vesicles were restricted to the perinuclear region (Fig. 4g). Therefore, we propose that Rab1a acts downstream of Nod1-Rip2 to drive insulin trafficking.

### Lysozyme-digested DAP-type PGN fragments from intestinal microbes direct insulin distribution in beta cells

**Fig. 5:**
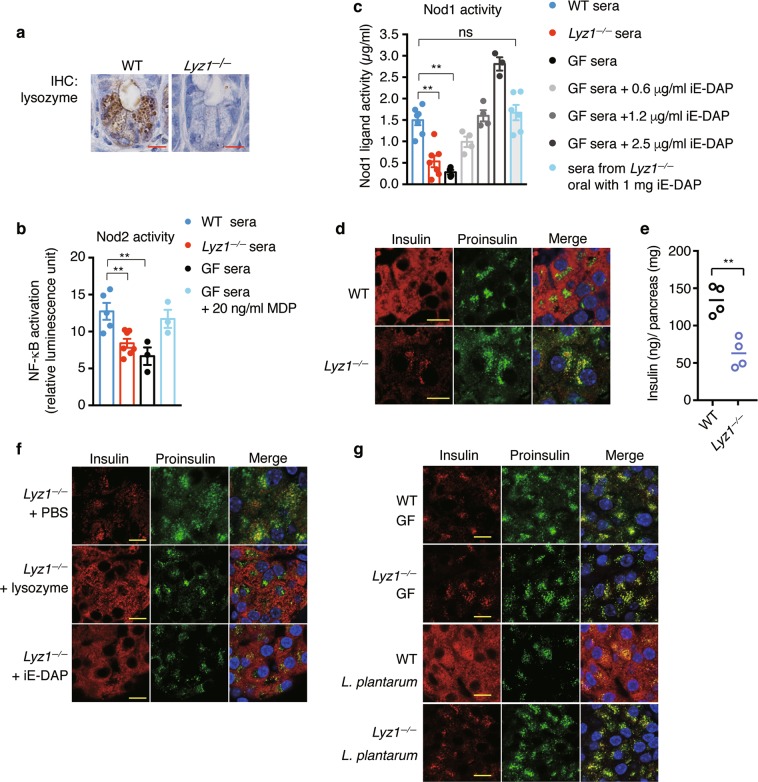
Lyz1 liberates microbial ligands from intestinal bacteria. **a** IHC staining of lysozyme in paraffin sections of ileal tissues from WT or *Lyz1*^*−/−*^ mice. **b** Nod2 ligand activity was measured in sera from WT, *Lyz1*^*−/−*^, or GF mice with HEK293-NOD2 reporter cells. GF sera supplemented with 20 ng/mL MDP was also included as the reference. Values represented as fold change relative to control empty vector in luciferase expression. Each symbol represents an individual animal, with columns presenting means ± s.e.m. **c** NOD1 bioactivity assay with a HEK-Blue mNOD1 reporter cells measuring Nod1 ligand activity in sera from WT, *Lyz1*^*−/−*^, GF and iE-DAP orally pretreated *Lyz1*^*−/−*^ mice. GF sera supplemented three different concentrations of iE-DAP were also included as the reference. Each symbol represents an individual animal, with columns presenting means ± s.e.m. **d** Immunostaining and confocal imaging of insulin and proinsulin in paraffin sections of pancrea from WT and *Lyz1*^*−/−*^ mice. **e** The amount of insulin in pancreatic tissues from WT and *Lyz1*^*−/−*^ mice. **f** Immunostaining and confocal imaging of insulin and proinsulin in paraffin sections of pancreata from *Lyz1*^*−/−*^ mice orally administrated with iE-DAP, lysozyme or mock-treated. **g** Immunostaining and confocal imaging of insulin and proinsulin in paraffin sections of pancreata from WT and *Lyz1*^*−/−*^ mice under germ-free conditions or mono-colonized with *L. plantarum* strain NY. Nuclei were counter-stained in blue (**d**, **f**, **g**). Scale bars, 10 μm in (**a**, **d**, **f**, **g**). Each symbol represents an individual animal, with bars presenting means. *P* values were calculated with a one-way ANOVA followed by Tukey’s post hoc tests (**b**, **c**), or a two-tailed Student’s *t-*test (**e**), **P < 0.01. Data are representative of three independent experiments

Small PGN fragments are constantly generated and actively recycled during bacterial wall synthesis and lysis.^37^ Only a small number of bacteria, such as *Bordetella pertussis* or *Vibrio fischeri*, which are defective in recycling, release small PGN fragments, such as tracheal cytotoxin (TCT), during their growth and replication.^38,39^ One study has shown that lysozyme in macrophages releases bacterial ligands from invading bacteria to activate Nod2.^40^ In the mouse genome, there are two lysozyme genes: lysozyme M or lysozyme 2, which is expressed in myeloid cells, and lysozyme P or lysozyme 1, which is mainly expressed in intestinal Paneth cells.^41^ Paneth cells, localized at the bottom of the intestinal crypts, secrete a large amount of Lyz1 into the intestinal lumen.^42^ Being the major lysozyme secreted into the gut lumen, Lyz1 is also generally referred to as intestinal lysozyme. We suspected that, with abundant bacteria present in intestine, Lyz1 might release Nod1 or Nod2 ligands from the bacterial wall by hydrolyzing bacterial PGN in the intestinal lumen. To test our hypothesis, we generated *Lyz1* knockout mice. In *Lyz1*^*−/−*^ mice, there was no lysozyme staining in Paneth cells (Fig. 5a). The expression of *Lyz2* mRNA was not significantly different in myeloid cells and Paneth cells in *Lyz1*^*−/−*^ and WT mice (Supplementary Information, Fig. S5a, b). We employed Nod1 and Nod2 bioactivity reporter assays to determine levels of circulating Nod1 and Nod2 ligands. Consistent with previous reports,^43,44^ sera from GF mice contained significantly lower activities of Nod2 and Nod1 ligands (Fig. 5b, c). Sera from *Lyz1*^*−/−*^ mice had significantly less activity of Nod2 ligands, at a level comparable with sera from GF mice (Fig. 5b). In our bioactivity assay, the Nod1 ligand activity in SPF mice was equivalent of approximately 1 μg/mL iE-DAP (Fig. 5c), comparable with a previous report.^43^ The concentrations we have used in this study to treat INS-1 or INS-1 832/13 were within the physiological range. Reduced activity of Nod1 ligands was present in sera from *Lyz1*^*−/−*^ mice compared to sera from WT mice (Fig. 5c). Supplementation with 1 mg iE-DAP by oral gavage restored the level of circulating Nod1 ligands in *Lyz1*^*−/−*^ mice to a level comparable with that in WT mice (Fig. 5c). The data suggest that Lyz1 contributes significantly to the level of PGN present in the circulation.

We next examined whether Lyz1 deficiency impacts insulin trafficking in beta cells. Lyz1 deficiency did not affect the overall expression of Nod1, Rip2, and Rab1a in islets (Supplementary Information, Fig. S5c–g). However, compared to insulin staining in islets from WT mice, insulin and proinsulin were co-localized to the perinuclear region in islets from *Lyz1*^*−/−*^ mice (Fig. 5d). The *Lyz1*^*−/−*^ mice were similar to GF mice, *Nod1*^*beta-cko*^, or *Rip2*^*beta-cko*^ mice in having a reduced amount of insulin in the pancreatic tissues (Fig. 5e). We treated *Lyz1*^*−/−*^ mice with recombinant lysozyme or iE-DAP via oral gavage. Either treatment restored the dispersed distribution pattern of insulin^+^ DCVs (Fig. 5f). These results are consistent with our hypothesis that Lyz1 is important for generating circulating Nod1 ligands that are sensed by beta cells to direct insulin trafficking.

To exclude changes in microbiota associated with the loss of intestinal lysozyme, we cohoused WT and *Lyz1*^*−/−*^ mice and examined their pattern of intracellular insulin distribution. We observed a perinuclear distribution pattern of insulin vesicles in the cohoused *Lyz1*^*−/−*^ mice whereas insulin vesicles remained dispersed throughout the cytosol in cohoused WT mice (Supplementary Information, Fig. S5h). Cohoused *Lyz1*^*−/−*^ mice had less insulin in the pancreatic tissues than WT mice (Supplementary Information, Fig. S5i). Thus, the difference of insulin distribution was not caused by possible changes in microbiota. To further exclude a possible confounding effect from complex microbiota, we derived GF *Lyz1*^*−/−*^ mice and inoculated them with a single bacterium, *L. plantarum*. Under GF conditions, insulin and proinsulin co-localized to the perinuclear region in beta cells in both WT and *Lyz1*^*−/−*^ mice (Fig. 5g). However, mono-colonization with *L. plantarum* restored insulin distribution in WT but not in *Lyz1*^*−/−*^ mice (Fig. 5g), supporting our hypothesis that Lyz1 is required for liberation of ligands from commensal microbe(s) to drive insulin trafficking in beta cells.

### Lysozyme-liberated Nod1 ligands recruit Nod1 onto beta cell DCVs and modulate insulin vesicle distribution

**Fig. 6:**
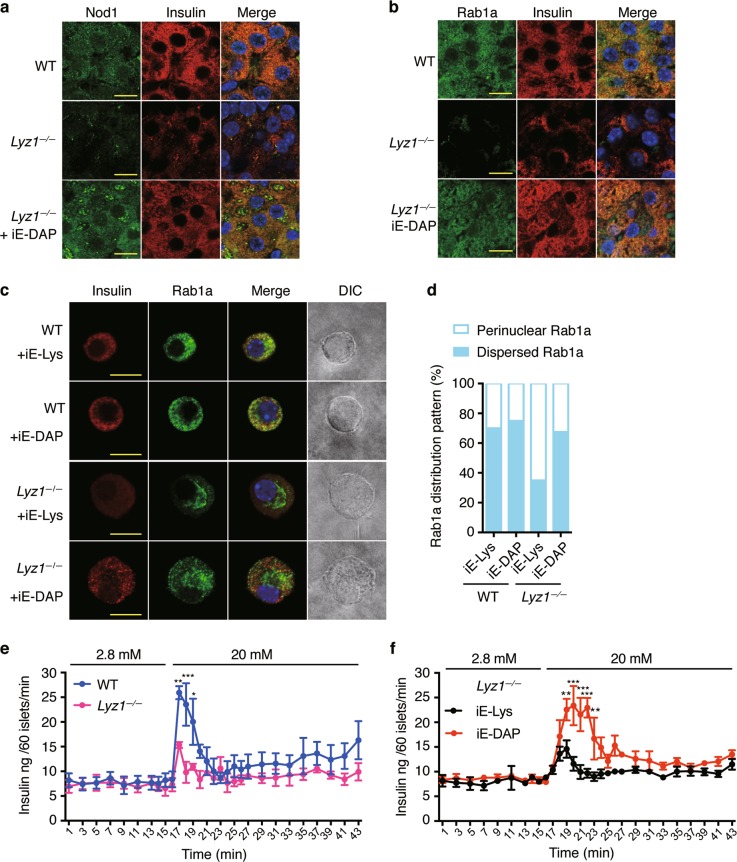
Deficiency of Lyz1 leads to defective insulin distribution in pancreatic beta cells. **a**, **b** Immunostaining and confocal imaging of Nod1 (**a**) or Rab1a (**b**) and insulin in paraffin sections of pancreata from WT, *Lyz1*^*−/−*^ mice or *Lyz1*^*−/−*^ mice orally administrated with iE-DAP. **c** Immunofluorescent staining and confocal imaging of Rab1a and insulin in primary cultured beta cells from WT or *Lyz1*^*−/−*^ mice, treated with iE-Lys or iE-DAP. **d** Quantification of Rab1a distribution, restricted in the perinuclear region (filled box) or dispersed (open box) in the cytosol as shown in **c**. 20 cells per condition were used for the analysis. **e** Measurement of ex vivo GSIS over time in islets from WT and *Lyz1*^*−/−*^ mice during perifusion with varying concentrations of glucose. *n* = 6. **f** Measurement of ex vivo GSIS over time in islets as treated during perifusion with varying concentrations of glucose. *n* = 6. Nuclei were counter-stained in blue (**a**–**c**). Scale bars, 10 μm in **a–c**. Symbols indicate mean values, with bars indicating s.e.m. *P* values were calculated with a two-tailed Student’s *t-*test (**e**), *P < 0.05, **P < 0.01, ***P < 0.001. Data are representative of three independent experiments

To determine whether Lyz1-liberated Nod1 ligands indeed recruit Nod1 and Rip2 onto DCVs to direct insulin trafficking, we first examined the cellular localization of Nod1 and Rip2 in beta cells in *Lyz1*^*−/−*^ mice. We found that Nod1 staining was much weaker on insulin^+^ and proinsulin^+^ DCVs in *Lyz1*^*−/−*^ mice compared with WT mice (Fig. 6a; Supplementary Information, Fig. S6a) whereas quantitative-PCR, IHC staining and immunoblotting in isolated islets showed that Nod1 levels were comparable in WT and *Lyz1*^*−/−*^ mice (Supplementary Information, Fig. S5c–e). This indicates that less Nod1 was recruited onto DCVs in *Lyz1*^*−/−*^ mice. The DCV localization of Rip2 and Rab1a in beta cells in *Lyz1*^*−/−*^ mice was similarly reduced (Fig. 6b; Supplementary Information, Fig. S6b). Treatment of *Lyz1*^*−/−*^ mice with oral iE-DAP restored Nod1, Rip2 and Rab1a localization on both insulin^+^ and proinsulin^+^ DCVs (Fig. 6a, b; Supplementary Information, Fig. S6b), indicating that Lyz1-liberated Nod1 ligands help Nod1, Rip2 and Rab1a translocate to DCVs.

To evaluate the direct effect of Nod1 ligands on insulin trafficking, we sought to determine whether Nod1 ligands may directly modulate Rab1a distribution in primary cultured beta cells. We chose to compare the effect of Nod1 ligands on beta cells from *Lyz1*^*−/−*^ and WT mice. We performed immunofluorescence staining on insulin and Rab1a in primary beta cells treated with iE-DAP or the inactive analog iE-Lys. IE-DAP treatment on beta cells from WT mice did not apparently alter the intracellular distribution of Rab1a, compared to iE-Lys treatment (Fig. 6c, d). Compared to dispersed Rab1a staining in beta cells from WT mice, Rab1a staining was restricted around nuclear region in beta cells from *Lyz1*^*−/−*^ mice (Fig. 6c, d). IE-DAP treatment induced the dispersed distribution of Rab1a in beta cells from *Lyz1*^*−/−*^ mice (Fig. 6c, d). The primary beta cells from *Lyz1*^*−/−*^ mice but not WT mice responded to iE-DAP treatment suggest that freshly isolated beta cells from WT mice contain sufficient Nod1 ligands to direct insulin trafficking.

We next sought to determine whether Nod1 ligands might affect insulin secretion with ex vivo GSIS assays. In ex vivo GSIS assays, isolated *Nod1*^*−/−*^ islets displayed impaired secretion compared to isolated WT islets (Supplementary Information, Fig. S6c). Similarly, isolated *Lyz1*^*−/−*^ islets displayed diminished GSIS compared to isolated WT islets (Supplementary Information, Fig. S6d) indicating bacteria-derived ligands were required for efficient GSIS. To further determine the direct effect of Nod1 ligands on the dynamics of insulin secretion from islets, we performed the GSIS assay with ex vivo perifusion. Compared to islets from WT mice, less insulin was secreted from islets from *Lyz1*^*−/−*^ mice upon 20 mM glucose stimulation (Fig. 6e). We wondered whether the diminished GSIS response in islets from *Lyz1*^*−/−*^ mice was indeed due to deficiency of circulating Nod1 ligands. To test this, we determined whether supplementing iE-DAP would restore the GSIS response in islets from *Lyz1*^*−/−*^ mice. Because the peri-islet basement membrane surrounding isolated islets may impede compound penetration, we chose to treat *Lyz1*^*−/−*^ mice in vivo and isolate islets afterwards. Islet perifusion assays on islets from *Lyz1*^*−/−*^ mice showed that iE-DAP treatment restored insulin secretion upon high glucose stimulation, compared to iE-Lys treatment (Fig. 6f). Collectively, our data demonstrate that Nod1 ligands modulate insulin intracellular distribution and affect insulin secretion from islets.

### Nod1/Rip2-directed insulin trafficking regulates glucose tolerance

Previous studies show that Nod1 ligands associate with insulin resistance and Nod1-deficient mice displayed normal glucose tolerance with normal chow diet.^6–9^ However, whether insulin secretion was affected in *Nod1*^*−/−*^ mice has not been determined previously. Indeed, we observed a normal glucose tolerance in *Nod1*^*−/−*^ mice (Supplementary Information, Fig. S7a, b). However, we noted the level of circulating insulin after glucose infusion in *Nod1*^*−/−*^ mice was significantly lower than that in WT mice (Supplementary Information, Fig. S7c). The total insulin content was also reduced in pancreata from *Nod1*^*−/−*^ mice compared to those from WT mice (Supplementary Information, Fig. S7d). Furthermore, our ex vivo GSIS assay has shown impaired insulin secretion in isolated islets from *Nod1*^*−/−*^ mice compared with those from WT mice (Supplementary Information, Fig. S6c). Therefore, *Nod1*^*−/−*^ mice have normal glucose tolerance but with reduced insulin secretion. The inconsistency between normal glucose tolerance and reduced insulin secretion in *Nod1*^*−/−*^ mice prompted us to determine whether insulin sensitivity may be altered in *Nod1*^*−/−*^ mice. The insulin tolerance test (ITT) assay showed *Nod1*^*−/−*^ mice displayed mildly enhanced insulin sensitivity compared to WT mice (Supplementary Information, Fig. S7e, f). Thus, *Nod1*^*−/−*^ mice had normal glucose tolerance, accompanied by decreased insulin secretion and enhanced insulin sensitivity.

**Fig. 7:**
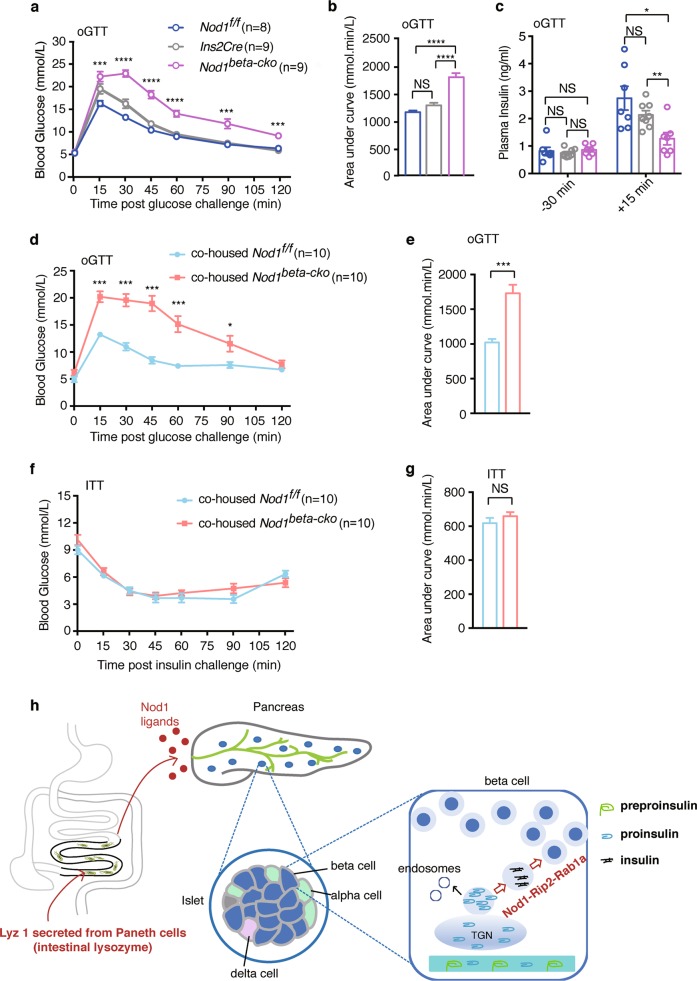
The Nod1 ligand-mediated crosstalk between intestine and islet is important for glucose tolerance. **a**, **b** Concentration of blood glucose during an oral GTT with 2 g/kg glucose in *Nod1*^*f/f*^, *Ins2Cre* and *Nod1*^*beta-cko*^ mice. **b** Area under the curve from **a**. **c** Concentration of plasma insulin in *Nod1*^*f/f*^, *Ins2Cre* and *Nod1*^*beta-cko*^ mice 30 min before and 15 min after oGTT. **d** Concentration of blood glucose during an oral GTT with 2 g/kg glucose in cohoused *Nod1*^*f/f*^ and *Nod1*^*beta-cko*^ littermates. **e** Area under the curve from **d**. **f** Concentration of blood glucose during an ITT in the cohoused *Nod1*^*f/f*^ and *Nod1*^*beta-cko*^ littermates. **g** Area under the curve from **f**. **h** Schematic of the intestine-islet crosstalk, in which intestinal lysozyme liberates Nod1 ligands from microbes into the circulation, and beta cells respond directly to the Nod1 ligands to direct intracellular insulin transport. Each symbol represents mean of individual animals in a group, and bars indicate s.e.m (**a**, **d**, **f**). Each symbol represents an individual animal, and lines or horizontal bars indicate median values (**c**). Data are representative of three independent experiments (**a**–**e**) and two independent experiments (**f**, **g**). *P* values were calculated with a two-way ANOVA followed by Tukey’s post hoc tests (**d**, **f**), one-way ANOVA followed by Tukey’s post hoc tests (**a**–**c**), a two-tailed Student’s t test (**e**, **g**). *P *<* 0.05, **P *<* 0.01, ***P *<* 0.001

To delineate the physiological function of Nod1 ligands on insulin secretion, we employed beta-cell-specific *Nod1 and Rip2* knockout mice. Specific loss of Rip2 in beta cells did not alter the size and number of the islets, or the size of beta cells in individual islets, compared to control mice (Supplementary Information, Fig. S7g–j). We performed oral glucose tolerance test (OGTT) to determine whether Nod1 or Rip2 deficiency in beta cells might affect glucose tolerance. *Nod1*^*beta-cko*^ mice had similar blood glucose levels as the control mice, *Nod1*^*f/f*^ and *Ins2Cre* mice, at the 16 h-fasting state (0 min in Fig. 7a). However, *Nod1*^*beta-cko*^ mice displayed significantly higher blood glucose levels during OGTT (Fig. 7a, b). Furthermore, the surge in plasma insulin concentration 15 min following glucose challenge was significantly attenuated in *Nod1*^*beta-cko*^ mice compared to control mice (Fig. 7c). A similar defect in glucose tolerance was observed in *Rip2*^*beta-cko*^ mice (data not shown). Intraperitoneal GTT assays also showed impaired glucose tolerance in *Rip2*^*beta-cko*^ mice (Supplementary Information, Fig. S7k, l) and *Nod1*^*beta-cko*^ mice (data not shown) compared to control mice. Thus, specific ablation of Nod1 or Rip2 in beta cells impaired glucose tolerance.

Since insulin insufficiency or peripheral insulin resistance can lead to glucose intolerance, we performed the insulin tolerance test (ITT) on *Nod*^*beta-cko*^ or *Rip2*^*beta-cko*^ and control mice. *Nod1*^*beta-cko*^ (Fig. 7f, g) and *Rip2*^*beta-cko*^ (Supplementary Information, Fig. S7m, n) mice were not more insulin resistant compared with control mice, excluding the possibility that the glucose intolerance in *Nod1*^*beta-cko*^ and *Rip2*^*beta-cko*^ mice was due to insulin resistance. Because intestinal microbial composition may modulate insulin sensitivity, we cohoused *Nod1*^*beta-cko*^ and *Nod1*^*f/f*^ littermates and performed GTT and ITT assays on them. We found that cohoused *Nod1*^*beta-cko*^ mice had impaired glucose tolerance compared to cohoused control mice (Fig. 7d, e) whereas insulin sensitivity in cohoused mice remained comparable (Fig. 7f, g). Thus, the defect in glucose tolerance in *Nod1*^*beta-cko*^ mice was not due to altered intestinal microbiota. Some studies have suggested that *Ins2Cre* mice may have altered glucose tolerance and insulin secretion;^45^ therefore, we performed GTT and ITT assays on *Ins2Cre* mice. Our experiments showed that *Ins2Cre* mice were comparable in glucose tolerance and insulin tolerance to *Rip2*^*f/f*^ mice (Supplementary Information, Fig. S7k–n). Therefore, direct sensing of Nod1 ligands in beta cells is critical for glucose-stimulated insulin secretion during GTT and is thus important for glucose tolerance.

We wanted to know whether ablating Nod1 sensing in beta cells may exacerbate hyperglycemia during DIO. *Nod1*^*f/f*^ and *Nod1*^*beta-cko*^ mice had similar weight gains during 8 weeks on a high-fat diet (HFD, Supplementary Information, Fig. S8a). In HFD-treated *Nod1*^*beta-cko*^ mice, glucose intolerance was exacerbated compared to HFD-treated control mice (Supplementary Information, Fig. S8b, c) and less insulin was secreted upon glucose challenge in vivo (Supplementary Information, Fig. S8d). HFD-treated *Nod1*^*beta-cko*^ mice had higher blood glucose levels than HFD-treated control mice at 6 h after starvation (Supplementary Information, Fig. S8e). Despite the higher glucose levels, HFD-treated *Nod1*^*beta-cko*^ mice had lower plasma insulin (Supplementary Information, Fig. S8f) suggesting a deficiency of circulating insulin. The exacerbated glucose intolerance and hyperglycemia in HFD-treated *Nod1*^*beta-cko*^ mice was not due to insulin resistance, because HFD-treated Nod1 *Nod1*^*beta-cko*^ mice were not more insulin resistant than the control mice (Supplementary Information, Fig. S8g). Thus, the lack of Nod1-mediated gut-islet crosstalk further exacerbates hyperglycemia associated with DIO.

## Discussion

We report an intestine-islet axis in which signals from commensal bacteria are sensed directly in islet beta cells to modulate insulin output (Fig. 7h). In the intestine-islet axis, Nod1, in the beta cells, senses the bacterial ligands released from commensal bacteria by Lyz1 in the intestinal lumen. Upon ligand binding, Nod1 and Rip2 recruit Rab1a onto insulin vesicles to drive insulin intracellular trafficking and promote insulin secretion. Defects in the intestine-islet axis lead to reduced glucose tolerance in the mouse. Thus, our study unveils a physiological role of Nod1 ligand-mediated crosstalk between intestine and islets to promote glucose homeostasis.

Bacterial Nod1 ligands are known to be involved in pathogenesis of insulin resistance in the murine model of DIO, which has been attributed to activating innate immunity.^6–9^ Our study and previous studies are complementary but not contradictory. Previous studies investigated the role of Nod1 ligands on insulin sensitivity in the settings of DIO or LPS administration.^6,8,9^ Previous studies did not address whether Nod1 ligands affected insulin secretion from islet beta cells in the physiological setting. Notably, *Nod1*^*−/−*^ mice had normal glucose tolerance on normal chow, in our study, as well as in a previous study.^6^ We found that the normal glucose tolerance in *Nod1*^*−/−*^ mice was accompanied by a reduced level of circulating insulin during GTT and impaired insulin secretion during GSIS. We hypothesized that the low level of Nod1 ligands in WT mice on normal chow might produce relative insulin resistance. Indeed, previously, *Schertzer* showed that Nod1 ligands directly reduced insulin sensitivity in isolated primary hepatocytes at a concentration as low as 100 ng/mL of iE-DAP in a Nod1-dependent manner.^7^ We found that the level of Nod1 ligands in SPF mice was equivalent to approximately 1 μg/mL of iE-DAP, and thus in a similar range to a previous report.^43^ It is possible that Nod1 ligands circulating in blood in WT mice on normal chow was sufficient to produce relative insulin resistance. Indeed, we found that *Nod1*^*−/−*^ mice were more insulin sensitive than WT mice. Thus, we postulated that the normal glucose tolerance observed in *Nod1*^*−/−*^ mice was a net result of reduced insulin secretion from islets and relatively enhanced insulin sensitivity. We propose that the act of Nod1 ligands on islets provides a critical mechanism to counterbalance the increased demand of insulin, associated with microbial colonization and increased levels of microbial products. Previous studies have used germline Nod1 knockout mice, which made it difficult to detect the effect of Nod1 ligands on insulin secretion. Our study, together with previous studies, argues for the counterbalancing effects of Nod1 ligands in the physiological vs pathological settings. In the setting of Diet Induced Obesity (DIO), the excess of Nod1 ligands in circulation may produce overt inflammation, leading to pronounced insulin resistance.

In obesity and in most situations of insulin-resistance, such as DIO, beta cells compensate for insulin resistance for a period of time by increasing secretory capacity and hyperplasia. The exacerbated hyperglymia in *Nod1*^*beta-cko*^ mice is consistent with our proposed mechanism that Nod1 sensing in beta cells is critical in blood sugar control, but does not necessarily support that Nod1 sensing in beta cells as a part of the compensatory response involved in HFD. We note: (1) supplementation of WT mice with iE-DAP did not change the cellular distribution of insulin in beta cells; (2) treatment of primary beta cells isolated from WT mice with iE-DAP did not change the recruitment of Rab1a onto insulin vesicles; (3) the effects of iE-DAP on insulin, Nod1, or Rab1a were only observed in cells deprived of Nod1 ligands, such as INS-1, INS-1 832/13, primary beta cells isolated from *Lyz1*^*−/−*^ mice, and mice deprived of Nod1 ligands, such as GF mice or *Lyz1*^*−/−*^ mice. These data argue that Nod1 ligands present in WT mice are sufficient for mediating the crosstalk, and additional Nod1 ligands can not further enhance the insulin production. Thus, our proposed mechanism does not contribute to the compensatory response during HFD.

Interestingly, opposing effects on insulin sensitivity and insulin secretion are not unprecedented. The chronic activation of IL-1β has been associated with the development of metabolic syndromes.^46^ However, postprandial macrophage IL-1β promotes insulin secretion, arguing for a physiological modulating role of IL-1β on whole-body glucose homeostasis.^47^ Situations associated with chronic inflammation might break the delicate balance on insulin sensitivity and insulin secretion mediated by IL-1β and Nod1 ligands, leading to overt insulin resistance and hyperglycemia. To understand the complex effects of bioactive molecules, such as IL-1β and Nod1 ligands, on metabolism, investigating their specific roles on individual tissues in the pathological and physiological settings is warranted.

In our study, we attributed the different distribution patterns of insulin trafficking in beta cells in WT vs GF, *Lyz1*^*−/−*^, *Nod1*^*−/−*^, or *Rip2*^*−/−*^ to the insufficiency of Nod1 ligands or failure to respond to Nod1 ligand because supplementation of iE-DAP sufficiently restored insulin distribution in GF and *Lyz1*^*−/−*^ mice. Our data argue for a direct effect of Nod1 ligands on beta cells. First, beta cell-specific ablation of Nod1 or Rip2 led to disrupted insulin distribution; second, treatment of iE-DAP on INS-1 or primary beta cells from *Lyz1*^*−/−*^ mice affected insulin trafficking; third, islets from *Lyz1*^*−/−*^ mice secreted significantly less insulin compared to islets from WT mice in ex vivo GSIS and treatment of iE-DAP rescued the insulin secretion in islet perifusion assays. Therefore, the robust insulin secretion in GSIS observed in WT mice depends on the availability of Nod1 ligands present in circulation in WT mice. Reduced levels of circulating Nod1 ligands in *Lyz1*^*−/−*^ mice led to diminished GSIS. Thus, our data reveal that bacterial Nod1 ligands are involved in mediating a robust insulin secretion response to glucose stimulation.

In our study, we focus on the gross changes of the cellular distribution of insulin vesicles modulated by intestinal Nod1 ligands. Mechanistically, we found that Nod1 and Rip2 recruited Rab1a onto DCVs and knockdown of Rab1a led to insulin vesicles retained around nuclei. Thus, we postulated that intestinal Nod1 ligands directed insulin vesicle distribution throughout the cytoplasm in a Rab1a-dependent manner. Previous studies have shown an elevated glucose level helps insulin vesicles to move along the microtubules in a manner that is largely dependent on kinesin-1 in cultured cells.^48,49^ Interestingly, Rab1a has been shown to modulate anterograde trafficking of melanosomes along the microtubules by recruiting kinesin-1.^33,35^ Thus, it is possible that Nod1 ligands direct the cytosolic distribution of insulin vesicles by promoting their movement along the microtubules. Of course, it cannot be excluded that Nod1 ligands may also affect the subsequently steps involved in exocytosis, such as docking, priming and fusion after insulin vesicles arrive at plasma membrane. We believe that the possibility warrants further studies.

In our study, we noted that a lack of intestinal Nod1 ligands, or deficiency of Nod1 or Rip2, all resulted in marked changes in three aspects, (1) the impaired cytosolic distribution of insulin vesicles, and (2) the impaired segregation of insulin and proinsulin, (3) the reduced amounts of insulin content. Insulin has been studied as a model cargo of DCVs for past decades. Currently, it is believed that insulin conversion and vesicle transport across the cytoplasm are likely coupled during insulin intracellular trafficking. Proinsulin positive immature DCVs bud off from TGN and undergo several deeply intertwined maturation steps including the homotypic fusion of immature DCVs, removal of certain soluble and transmembrane cargos, cargo proteotic process and cargo condensation. Our observation that reduced amounts of insulin accompany impaired proinsulin to insulin conversion and antetrograde trafficking may imply that impaired proinsulin to insulin conversion may lead to removal of insulin and proinsulin from maturing DCVs. Our results support the notion that insulin conversion and vesicle transport are deeply coupled. Further studies are warranted to uncover the molecular mechanisms by which the Nod1-Rip2-Rab1a axis drives insulin vesicle movement and through which insulin conversion and cytosolic distribution are coupled.

Nod1 and Nod2 ligands have been found to play important roles in modulating a range of host physiological responses in the mutually beneficial interactions between microbe and host, including inducing lymphoid tissue genesis,^50^ maintaining Goblet cells,^51,52^ promoting mucosal adjuvant activity of cholera toxin,^53^ and inducing expression of hematopoietic cytokines by mesenchymal stromal cells to promote hematopoiesis.^44^ How the Nod1 and Nod2 ligands are liberated from the bacterial wall was previously unknown. Lysozyme is an endo-*N*-acetylmuramidase which cleaves the beta-1,4-glycosidic bonds between *N*-acetylmuramic acid (Mur*N*ac) and *N*-acetylglucosamine (Glc*N*ac) that form the backbone of PGN. We suspect that lysozyme may break the backbone of the interconnected PGN chains, after which peptidoglycan hydrolases from microbes and the host may further digest PGNs into small fragments capable of entering the circulation. Along this line, one previous study showed that a secreted bacterial peptidoglycan hydrolase from *Enterococcus faecium* generates PGN fragments that protect *C. elegans* against *Salmonella* pathogenesis through *tol-1* signaling,^54^ indicating that hydrolytic enzymatic activities are required to liberate PGN fragments from bacterial cell walls. Of note, bacteria release small Nod1 ligand molecules containing iE-DAP during cell division independently of host enzymes,^55^ and the crowded community of intestinal microbes may recycle the free peptidoglycans, thus limiting the total availability amount of Nod1 ligands for host uptake. Furthermore, a higher level of Nod1 ligands was present in the sera from *Lyz*1^*−/−*^ mice than GF mice, also suggesting the existence of lyz-1-independent release of Nod1 ligands from the bacterial cell wall. Taken together, intestinal lysozyme may increase the amount of PGNs released from the bacterial cell wall and contribute to the levels of circulating Nod1 ligands. Whether intestinal lysozyme is involved in the broader host-microbe crosstalk remains to be determined organ by organ.

In summary, Nod1 ligands from intestinal microbes directly modulate insulin trafficking in beta cells, which increases insulin output to meet systemic demand. Lyz1 modulates the levels of bacterial ligands in the circulation and this may have broad implications for the host-microbe association. Thus, our study uncovers a host-microbe collaboration to generate microbial signals and reveals that intestinal-islet crosstalk is part of the mutualistic interaction between host and microbes.

## Materials and methods

### Mice

*Nod2*^*−/−*^ and *Myd88*^*−/−*^ mice on a C57BL/6J background were described previously.^25^ *Lyz1*^*−/−*^, *Nod1*^*−/−*^, *Rip2*^*f/f*^, and *Nod1*^*f/f*^ mice were generated using the CRISPR/Cas9 method. *Ins2Cre* (Stock number 003573) mice were from Jackson Laboratory. 8-12-week-old gender-matched mice were used in the study. Mice were housed at 2-5 animals per cage in a 12-h light/12-h dark cycle with ad libitum access to food and water at a controlled temperature (23 °C ± 2 °C). All SPF mice including WT C57BL/6J were bred and housed in an AAALAC-accredited barrier facility for specific pathogen-free (SPF) mice. For co-housing experiments, littermates were used and cohoused after weaning before experimentation.

For germ-free (GF) mice, WT C57BL/6J mice were bred and maintained in a germ-free mouse facility in sterile isolators and screened for bacterial, fungal, and viral contamination.^25^ A breeding colony of GF *Lyz1*^*−/−*^ mice was established by cesarean section and foster nursing. Adult GF mice were conventionalized by feeding with cecum contents obtained from SPF mice using gastric gavage once per day for three consecutive days and maintained in isolators for at least 3 weeks afterwards.

All animal protocols were approved by the Committee of Institutional Animal Care and Research.

### Generation of germline knockout mice with the CRISPR/Cas9 technique

sgRNA oligos targeting *Lyz1* and *Nod1* were designed using the CRISPR design tool (http://crispr.mit.edu) against the mm9 mouse genome assembly. Potential off-target sites were identified with ungapped alignment, allowing for up to four mismatches in the target sgRNA sequences. The sgRNA oligo pairs listed in Supplementary Information, Table S1 were synthesized (Invitrogen) and used to construct sgRNA-expressing vectors. The sgRNA-expressing vector used was PUC57-sgRNA.^56^ Synthesized sgRNA oligos were denatured at 95 °C for 5 min and annealed at room temperature, before being cloned between two BsaI sites of a linearized PUC57-sgRNA expression vector. The sgRNA expression vectors were then linearized by DraI and transcribed in vitro using a MEGAshortscript T7 kit (Thermo Fisher, AM1354). The pST1374-Cas9 vector was linearized using Age1 enzyme and transcribed in vitro using a T7 Ultra Kit (Thermo Fisher, AM1345). Both the Cas9 mRNA and the sgRNAs were purified using a MEGAclear kit (Thermo Fisher, AM1908) and eluted in RNase-free water.

*Lyz1* or *Nod1* mutant mice were generated by injection of in vitro transcribed sgRNAs (50 ng/μL) and Cas9 mRNA (100 ng/μL) into the cytoplasm of pronuclear stage C57BL/6J zygotes (Laboratory Animal Research Center, Tsinghua University) in M2 medium (Sigma). Approximately 100 zygotes were injected and subsequently transferred to the oviduct of pseudo-pregnant B6CBAF1 females, from which viable founder mice were obtained.

The target sites of the viable founder mice were amplified by PCR with specific primer pairs and sequenced (primers listed in Supplementary Information, Table S1). *Lyz1* and *Nod1* mutant founders were identified. Subsequently, the potential off-target sites in founder mice were amplified with specific primers (Supplementary Information, Table S1) and sequenced. No off-target mutation was found in F1 mice.

### Generation of conditional knockout mice using Cre/loxP and CRISPR/Cas9 technique

The service to generate the *Rip2*^*f/f*^ and *Nod1*^*f/f*^ founder lines was provided by Casgene Biotech Company (Beijing, China). Briefly, for Rip2 targeting, a sgRNA oligo was designed to target a region upstream of exon 2 (Supplementary Information, Fig. S1). A circular donor vector with Rip2 exon2 flanked by loxP sites and homology arms was used as a template to repair the double-strand break (DSB) by homologous recombination (Supplementary Information, Fig. S1). In the donor vector, the loxP site was located downstream of the protospacer adjacent motif (PAM) of the sgRNA-targeting site. For Nod1 targeting, a sgRNA oligo was designed to target a region upstream of exon 3 (Supplementary Information, Fig. S2). A circular donor vector with Nod1’s exon 3 flanked by loxP sites and homology arms was used as a template to repair the DSB by homologous recombination (Supplementary Information, Fig. S2). In both donor vectors, the loxP site was located downstream of the protospacer adjacent motif (PAM) of the sgRNA-targeting site. Pronuclear stage C57BL/6J zygotes were injected with sgRNA, circular donor vectors and Cas9 mRNA, and subsequently transferred to the oviduct of pseudo-pregnant B6CBAF1 females, from which viable founder mice were obtained.

The target sites of the viable founder mice were amplified by PCR with specific primer pairs and sequenced (Supplementary Information, Table S1). *Rip2*^*f/+*^ and *Nod1*^*f/+*^ founders were identified. Beta-cell-specific knockout mice (*Rip2*^*beta-cko*^ or *Nod1*^*beta-cko*^) were obtained by crossing a conditional allele to *Ins2cre* mice.

### Cell lines and cultures

HEK293T cells were cultured in DMEM medium (Hyclone) supplemented with 10% fetal bovine serum (FBS, Hyclone) and 1% Pen/Strep (Gibco). HEK-blue™ mNOD1 cells (InvivoGen, hkb-mnod1) and HEK/mNod2 cells (InvivoGen, 293-mnod2) were cultured in DMEM (Hyclone) supplemented with 10% FBS (Hyclone), 2 mM L-glutamine (Gibco), 50 U/mL penicillin (Gibco), 50 μg/mL streptomycin (Gibco), 100 μg/mL Normocin (InvivoGen), 30 μg/mL blasticidin (InvivoGen), and 100 μg/mL Zeocin (InvivoGen).

INS-1 (National Infrastructure of Cell Line Resources, China) or INS-1 832/13 cells (gift from Dr. Yong Liu from Wuhan Univ.) were cultured in RPMI-1640 supplemented with 10% FBS, 1 mM sodium pyruvate, 10 mM HEPES, 50 μM 2-mercaptoethonal, 100 unit/mL penicillin and 100 μg/mL streptomycin. All the cells were kept at 37 °C in a humidified 5% CO_2_ incubator.

INS-1 cells were grown on cover slips and were kept in complete RPMI 1640 medium for 72 h,^29^ then treated with iE-DAP (1 μg/mL) or iE-Lys for 20 h in RPMI 1640 medium containing 3 mM glucose before immunofluorescence staining.

### Isolation and primary culture of mouse pancreatic beta cells

Islets were isolated as described.^57^ For preparation of primary beta cells, 150 isolated islets from each mouse were handpicked into a 24-well plate and incubated with 0.3 mL cell dissociation reagent (StemPro® accutase® Gibico) for about 2 min at 37 °C to break down islets into single cells before adding 1 mL culture medium to stop the digestion. The mixture was centrifuged at 450 g for 5 min and the cell pellet was resuspended in RPMI 1640 culture medium (10% FBS, HEPES 10 mM, glutamine 2 mM, glucose 5.6 mM). 10^5^ primary beta cells were seeded into each well in a 24-well plate or seeded onto cover clips for further analysis.

### Plasmids

Murine Rip2 cDNA was reverse transcribed from mRNA prepared from mouse intestinal crypts and cloned into a pcDNA3-HA vector. The pcDNA3-HA vector was derived from pcDNA3.1(+) by inserting a HA tag sequence between the BamH1 and EcoR1 sites on the original vector. Murine Rab1a cDNA was reverse transcribed from mRNA prepared from mouse lung and cloned into the pCMV-myc vector. Sequences were confirmed by Sanger sequencing.

### Bacterial ligands or recombinant lysozyme supplementation to mice

All bacterial products were dissolved in endotoxin-free PBS. 8-10 week-old male mice were orally administered with MDP (0.5 mg/mouse), LPS (0.5 mg/mouse), iE-DAP (1 mg/mouse), Tri-DAP (1 mg/mouse), PGN (0.5 mg/mouse) or PBS (sham) twice, 12 h apart. 4 h after the second treatment, mice were sacrificed and pancreatic tissues were collected.

Wild-type and *Lyz1*^*−/−*^ littermates were fed with recombinant lysozyme (100 mg/kg) in the same manner as described above.

### Mono-colonization with Lactobacillus plantarum or Lactococcus lactis

*Lactobacillus plantarum* strains NY and GY7 were obtained from Dr. Jin Zhong, and *Lactococcus lactis* strain NZ9000 was from Dr. Wei Chen. *L. plantarum* was cultured in MRS broth, and *L. lactis* was cultured in GM17 medium as described.^58^ Individual GF mice were gavaged with 100 μL (1 × 10^7^ CFU) of each strain, feces were collected after inoculation and subjected to morphological examination under a microscope. Bacterial DNA was extracted from collected feces, and primers (listed in Supplementary Information, Table S1) specific for *L. lactis* and *L. plantarum* were used for PCR identification.^59,60^

### Oral glucose tolerance test and intraperitoneal glucose tolerance test

OGTT was performed by oral gavage of glucose (2 g/kg as a 20% solution) and ipGTT was performed by injecting glucose (2 g/kg as a 20% solution) intraperitoneally in 16-h-fasted mice at about 12 weeks of age. Blood was collected by tail snip before (0 min) or 15, 30, 45, 60, 90, and 120 min after glucose administration. The blood glucose level was determined using a GlUCOCARD blood glucometer and Test Strip II (Arkray).

### Intraperitoneal insulin tolerance test

6 h-fasted 12-week-old mice were injected intraperitoneally with a solution of insulin (0.5 IU/kg) and the blood glucose level was measured before (0 min) and 15, 30, 45, 60, 90, and 120 min after the injection using a GLUCOCARD blood glucometer and Test Strip II (Arkray).

### Determination of pancreatic insulin and proinsulin content

After 16 h of fasting, mice were euthanized by CO_2_, and whole pancreata were dissected and immediately frozen in liquid nitrogen. Protein extracts were prepared using the acid-ethanol method.^61,62^ In brief, pancreata weighing 200–300 mg were homogenized in 15 mL acid-ethanol (95% ethanol and 10.2 N HCl at a ratio of 50:1) using a homogenizer for 2 min. After an overnight incubation at 4 °C, the extracts were centrifuged at 650 × g for 30 min at 4 °C. Insulin concentrations in pancreatic extracts were measured via ELISA (Insulin Kit from Millipore, proinsulin kit from Alpco), following the manufacturer’s instructions.

### Ex vivo glucose-stimulated insulin secretion assay with perifusion or incubation

Islets of WT or *Lyz1*^*−/−*^ mice, or *Lyz1*^*−/−*^ mice oral gavaged with 1 mg iE-DAP or equal molar concentration of iE-Lys, were isolated and incubated in RPMI1640 complete medium overnight. For perifusion glucose-stimulated insulin secretion (GSIS), 60 handpicked islets from each animal were placed into Bio-Gel P-4 bead chambers for parallel perfusion GSIS analysis. An automated perfusion system (Bio rep® Perifusion System; BioRep) was employed for the assay. Islets were first equilibrated in Krebs-Ringer bicarbonate HEPES buffer (KRBH, NaCl 128 mM, KCl 4.8 mM, KH_2_PO_4_ 1.2 mM, MgSO_4_ 1.2 mM, CaCl_2_ 2.5 mM, NaHCO_3_ 5 mM, HEPES 10 mM, fatty acid-free BSA 0.1%) containing 2.8 mM glucose for 30 min and then perfused at a flow rate of 0.1 mL/min using the following sequential buffers, KRBH with 2.8 mM glucose for 10 min, KRBH with 20 mM glucose for 35 min. The perifusate was collected every minute in an automatic fraction collector.

For GSIS with batch incubation, islets isolated from WT and *Nod1*^*−/−*^ mice were incubated in RPMI1640 complete medium overnight. 80 islets were handpicked for each animal and transferred into a 6-well plate. Islets were first equilibrated in 2.8 mM glucose KRBH for 30 min, then manually and sequentially transferred to 2.8 mM KRBH for 30 min and then 16.7 mM glucose KRBH for another 30 min. INS-1 832/13 cells were seeded at a density of 5 × 10^5^ cells/well and grown in 6-well plate at 37 °C and 5% CO_2_ in a humidified atmosphere to 90% confluence over 3 days prior to experimentation. Before the GSIS assay, cells were preincubated in supplemented RPMI containing 3 mM glucose, 1 μg/mL iE-Lys or iE-DAP for 20 h. For GSIS assay, culture media was replaced with 2 mM glucose KRBH for 30 min, after which the buffer was replaced with 10 mM glucose KRBH for 30 min followed by stimulation with 30 mM KCl KRBH for 30 min. At each end of stimulation period, samples were collected for insulin measurement by ELISA.

### INS-1 cells or primary beta cells immunofluorescence staining

Cells grown on cover slips were washed with cold PBS, fixed in 4% PFA for 15 min at 4 °C, washed with cold PBST (PBS with 0.1% Triton X-100) for 3 times. After being blocked with 10% goat sera in PBST, cells were incubated with primary antibodies at 4 °C overnight and subsequently fluorophore-conjugated secondary antibodies at RT for 2 h. Between each incubation step, cover slips were washed with PBST three times for 5 min. Normal goat serum blocking reagent was present throughout all antibody incubation steps. Cover slips were then counterstained with DAPI and mounted in Fluoromount-G. Confocal images were obtained with Zeiss LSM 700 or Zeiss LSM 710 imaging systems under ×63 oil objectives.

The distribution patterns of Rab1a in primary cultured beta cells were quantified blindly. The staining patterns of Rab1a were either cytosolic dispersed or concentrated around nuclei (perinuclear). The distribution of Rab1a in at least 20 cells for each condition was analyzed.

The colocalization of Nod1 and Insulin was analyzed using Pearson’s correlation coefficient with ImageJ (NIH version 2.0.0).

### Paraffin-embedded tissue immunostaining

Pancreata were collected from mice overnight fasted (appta 16 h). Formalin-fixed, paraffin-embedded tissues (pancreata  and small intestines) were subjected to histological examination following either immunofluorescence staining or immunohistochemistry staining as described.^25^ Briefly, tissue slices (6 μm in thickness) were mounted on positively charged glass and dewaxed. Antigen retrieval was performed by incubation in 0.01 M sodium citrate buffer (pH 6.0) for 25 min in a boiling steamer.

For immunofluorescence staining, slides were then blocked with normal goat serum blocking reagent for 30 min, followed by sequential incubation with primary antibodies at 4 °C overnight and fluorophore-conjugated secondary antibodies at RT for 2 h. Slides were then counterstained with DAPI and mounted in Fluoromount-G. Confocal images were obtained with Zeiss LSM 700 or Zeiss LSM 710 imaging systems.

For immunohistochemistry staining, slides were then performed with an UltraSensitive ^TM^ SP Kit. After sequentially incubation with hydrogen peroxide (approcimately 15 min), blocking serum (~20 min), primary antibodies (overnight at 4 °C), biotinylated secondary antibody (~20 min) and streptavidin-peroxidase (~20 min), immunoreactivity was visualized using diaminobenzidine (DAB). The slides were counterstained with hematoxylin, mounted, and observed with a light microscope (Nikon Eclipse 90i).

### Stable shRNA knockdown of Rab1a in INS-1 cells

The pSuper RNAi system was used to establish the stable Rab1a knockdown INS-1 cells. Rab1a-targeting oligos (KD #1 and KD #2, sequences in Supplementary Information, Table S1) or control oligo (scramble, sequence in Supplementary Information, Table S1) were prepared by annealing a pair of oligonucleotides. The annealed oligos were then cloned into the *Bgl*II and *Hin*dIII sites of the pSuper.Retro.Puro vector following the manufacturer’s instruction. HEK293T cells were used as a packaging cell line to produce retroviral supernatants. INS-1 cells were transduced with the retroviral supernatants after the addition of 6 μg/mL polybrene for 3 h and allowed to recover for 24 h with fresh medium. Transduced cells were selected with 2 μg/mL puromycin for 72 h. Stable knockdown cell clones were established and knockdown efficiency were determined by Q-RCR and immunoblotting.

### Serum Nod1/2 ligand bioactivity assay

For the Nod1 ligand bioactivity assay, HEK-blue™ mNOD1 293 cells (InvivoGen) were cultured according to manufacturer’s guidance. Cells were seeded 40,000 cells/well in a 96-well plate in 200 μL complete DMEM medium overnight. 2 μL mouse serum (65 °C 5 min heat inactivated) diluted with 18 μL PBS, or iE-DAP of different concentration in 20 μL PBS as standard was mixed with 180 μL HEK-Blue™ Detection medium (InvivoGen), respectively. Once samples and standard are prepared, all old media was discarded from 96-well plates, and then 200 μL of sample or standard solutions added. The plate was incubated in the 37 °C, CO_2_ incubator for about 24 and absorbance read at 630 nm.

For the Nod2 ligand bioactivity assay, HEK293 cells stably expressing mouse Nod2 (InvivoGen) were cultured according to manufacturer’s guidance. Cells were seeded at a density of 3 × 10^5^ cells per well in a 24-well plate before transfection. Cells were transfected with 500 ng NF-κB-luc, 50 ng Renilla plasmid and 5 μL serum with Lipofectamine 2000, following the previous procedure.^43^ Luciferase expression was measured 24 h after transfection using a Luciferase Assay System (Promega, E2920) according to the manufacturer’s instructions.

### Co-immunoprecipitation (IP)

For co-IPs following overexpression, 2 × 10^6^ transfected HEK293T cells were harvested, washed with 1 mL cold PBS, and lysed in 400 μL cold IP buffer (50 mM Tris, 150 mM NaCl, 2 mM EDTA, 1% NP-40, 0.5 mM PMSF, protease inhibitor cocktail from Roche) on ice for 30 min. For the endogenous co-IP, 1 × 10^7^ INS-1 cells were harvested, washed with 1 mL cold PBS, and lysed in 600 μL cold IP buffer on ice for 30 min. Then cell nuclei and debris were removed by centrifugation at 10,000×*g* for 10 min at 4 °C. Supernatants (10 μL) were saved for input. The rest of the supernatants were precleared with 15 μL protein G-conjugated DYNA beads for 30 min at 4 °C. IP antibody was added to supernatants for 2 h at 4 °C before 15 μL DYNA beads were added to pull down the protein-antibody complexes for 1 h. The antibody complex bound to DYNA beads was washed three times with 400 μL IP buffer before being eluted with 30 μL SDS sample buffer (1×). Both inputs and eluents were subjected to immunoblotting analysis.

### Gene expression analysis using quantitative reverse-transcription PCR

RNA was extracted from isolated islets with TRIzol following the manufacturer’s instructions. mRNA was reverse transcribed using a PrimerScript RT reagent kit. Q-PCR reactions were then performed with SYRB Premix Taq on an ABI7500 thermal cycler in triplicate. The following thermal cycling conditions were used: 95 °C for 30 s, followed by 40 cycles of 95 °C for 5 s and 60 °C for 34 s. The specificity of Q-PCR was verified with melting curves for each PCR reaction. Primers specific for mouse *Gapdh* were used as control to normalize loading. The level of target mRNA was determined by Delta-Delta Ct values between the target and loading control. Primers for Q-PCR are listed in Supplementary Information, Table S1.

### Quantification and statistical analysis

All statistical analyses were conducted with GraphPad Prism version 7.0. For comparisons between two groups, significance was determined using the two-tailed Students’s *t*-test. For comparisons among more than two groups, a one-way or two-way ANOVA followed by Tukey’s post hoc tests. *P < 0.05, **P < 0.01. The data are representative of at least three experiments. The sample size and the replicate number of experiments are reported in the text, Figures and Figure Legends.

Antibodies and reagents used in this study were listed in the Supplementary Information, Table S2.

## Supplementary information

Supplementary information, Figure S1

Supplementary information, Figure S2

Supplementary information, Figure S3

Supplementary information, Figure S4

Supplementary information, Figure S5

Supplementary information, Figure S6

Supplementary information, Figure S7

Supplementary information, Figure S8

Supplementary information, Table S1

Supplementary information, Table S2

## References

[1] Backhed F (2004). The gut microbiota as an environmental factor that regulates fat storage. Proc. Natl Acad. Sci. USA.

[2] Backhed F, Ley RE, Sonnenburg JL, Peterson DA, Gordon JI (2005). Host-bacterial mutualism in the human intestine. Science.

[3] Backhed F, Manchester JK, Semenkovich CF, Gordon JI (2007). Mechanisms underlying the resistance to diet-induced obesity in germ-free mice. Proc. Natl Acad. Sci. USA.

[4] Schroeder BO, Backhed F (2016). Signals from the gut microbiota to distant organs in physiology and disease. Nat. Med..

[5] Rabot S (2010). Germ-free C57BL/6J mice are resistant to high-fat-diet-induced insulin resistance and have altered cholesterol metabolism. FASEB J..

[6] Amar J (2011). Intestinal mucosal adherence and translocation of commensal bacteria at the early onset of type 2 diabetes: molecular mechanisms and probiotic treatment. EMBO Mol. Med..

[7] Schertzer JD (2011). NOD1 activators link innate immunity to insulin resistance. Diabetes.

[8] Cavallari JF (2017). Muramyl dipeptide-based postbiotics mitigate obesity-induced insulin resistance via IRF4. Cell Metab..

[9] Chan KL (2017). Circulating NOD1 activators and hematopoietic NOD1 contribute to metabolic inflammation and insulin resistance. Cell Rep..

[10] Hussain MA, Akalestou E, Song WJ (2016). Inter-organ communication and regulation of beta cell function. Diabetologia..

[11] Perry RJ (2016). Acetate mediates a microbiome-brain-beta-cell axis to promote metabolic syndrome. Nature.

[12] Hill JH, Franzosa EA, Huttenhower C, Guillemin K (2016). A conserved bacterial protein induces pancreatic beta cell expansion during zebrafish development. Elife.

[13] Hou JC, Min L, Pessin JE (2009). Insulin granule biogenesis, trafficking and exocytosis. Vitam. Horm..

[14] Rutter GA, Hill EV (2006). Insulin vesicle release: walk, kiss, pause… then run. Physiology.

[15] Zhu X, Orci L, Carroll R, Norrbom C, Ravazzola M, Steiner DF (2002). Severe block in processing of proinsulin to insulin accompanied by elevation of des-64,65 proinsulin intermediates in islets of mice lacking prohormone convertase 1/3. Proc. Natl Acad. Sci. USA.

[16] Du W (2016). HID-1 is required for homotypic fusion of immature secretory granules during maturation. Elife.

[17] Jo J, Choi MY, Koh DS (2007). Size distribution of mouse Langerhans islets. Biophys. J..

[18] Kilimnik G, Kim A, Jo J, Miller K, Hara M (2009). Quantification of pancreatic islet distribution in situ in mice. Am. J. Physiol. Endocrinol. Metab..

[19] Caruso R, Warner N, Inohara N, Nunez G (2014). NOD1 and NOD2: signaling, host defense, and inflammatory disease. Immunity.

[20] Philpott DJ, Sorbara MT, Robertson SJ, Croitoru K, Girardin SE (2014). NOD proteins: regulators of inflammation in health and disease. Nat. Rev. Immunol..

[21] Kopp E, Medzhitov R (2003). Recognition of microbial infection by Toll-like receptors. Curr. Opin. Immunol.

[22] Vollmer W, Blanot D, de Pedro MA (2008). Peptidoglycan structure and architecture. FEMS Microbiol. Rev..

[23] Irving AT (2014). The immune receptor NOD1 and kinase RIP2 interact with bacterial peptidoglycan on early endosomes to promote autophagy and inflammatory signaling. Cell Host. Microbe..

[24] Nakamura N (2014). Endosomes are specialized platforms for bacterial sensing and NOD2 signalling. Nature.

[25] Zhang Q (2015). Commensal bacteria direct selective cargo sorting to promote symbiosis. Nat. Immunol..

[26] Asfari M (1992). Establishment of 2-mercaptoethanol-dependent differentiated insulin-secreting cell lines. Endocrinology.

[27] Hohmeier HE (2000). Isolation of INS-1-derived cell lines with robust ATP-sensitive K+ channel-dependent and -independent glucose-stimulated insulin secretion. Diabetes.

[28] Xu T (2014). The IRE1alpha-XBP1 pathway regulates metabolic stress-induced compensatory proliferation of pancreatic beta-cells. Cell Res..

[29] Lorenz MA, El Azzouny MA, Kennedy RT, Burant CF (2013). Metabolome response to glucose in the beta-cell line INS-1 832/13. J. Biol. Chem..

[30] Cao J (2014). Insight into insulin secretion from transcriptome and genetic analysis of insulin-producing cells of Drosophila. Genetics.

[31] Hickey AJ (2009). Proteins associated with immunopurified granules from a model pancreatic islet beta-cell system: proteomic snapshot of an endocrine secretory granule. J. Proteome Res..

[32] Brunner Y (2007). Proteomics analysis of insulin secretory granules. Mol. Cell Proteomics..

[33] Ishida M, Ohbayashi N, Maruta Y, Ebata Y, Fukuda M (2012). Functional involvement of Rab1A in microtubule-dependent anterograde melanosome transport in melanocytes. J. Cell Sci.

[34] Mukhopadhyay A (2011). Proteomic analysis of endocytic vesicles: Rab1a regulates motility of early endocytic vesicles. J. Cell Sci..

[35] Ishida M, Ohbayashi N, Fukuda M (2015). Rab1A regulates anterograde melanosome transport by recruiting kinesin-1 to melanosomes through interaction with SKIP. Sci Rep.

[36] Liu X (2016). Rab1A mediates proinsulin to insulin conversion in beta-cells by maintaining Golgi stability through interactions with golgin-84. Protein Cell.

[37] Vollmer W, Joris B, Charlier P, Foster S (2008). Bacterial peptidoglycan (murein) hydrolases. FEMS Microbiol. Rev.

[38] Cookson BT, Tyler AN, Goldman WE (1989). Primary structure of the peptidoglycan-derived tracheal cytotoxin of Bordetella pertussis. Biochemistry.

[39] Koropatnick TA (2004). Microbial factor-mediated development in a host-bacterial mutualism. Science.

[40] Davis KM, Nakamura S, Weiser JN (2011). Nod2 sensing of lysozyme-digested peptidoglycan promotes macrophage recruitment and clearance of S. pneumoniae colonization in mice. J. Clin. Invest..

[41] Markart P (2004). Comparison of the microbicidal and muramidase activities of mouse lysozyme M and P. Biochem. J.

[42] Bevins CL, Salzman NH (2011). Paneth cells, antimicrobial peptides and maintenance of intestinal homeostasis. Nat. Rev. Microbiol.

[43] Clarke TB (2010). Recognition of peptidoglycan from the microbiota by Nod1 enhances systemic innate immunity. Nat. Med..

[44] Iwamura Chiaki, Bouladoux Nicolas, Belkaid Yasmine, Sher Alan, Jankovic Dragana (2017). Sensing of the microbiota by NOD1 in mesenchymal stromal cells regulates murine hematopoiesis. Blood.

[45] Lee JY (2006). RIP-Cre revisited, evidence for impairments of pancreatic beta-cell function. J. Biol. Chem..

[46] Ballak DB, Stienstra R, Tack CJ, Dinarello CA, van Diepen JA (2015). IL-1 family members in the pathogenesis and treatment of metabolic disease: focus on adipose tissue inflammation and insulin resistance. Cytokine.

[47] Dror E (2017). Postprandial macrophage-derived IL-1beta stimulates insulin, and both synergistically promote glucose disposal and inflammation. Nat. Immunol..

[48] Pouli AE (1998). Secretory-granule dynamics visualized in vivo with a phogrin-green fluorescent protein chimaera. Biochem. J..

[49] Varadi A (2003). and cytoplasmic dynein orchestrate glucose-stimulated insulin-containing vesicle movements in clonal MIN6 beta-cells. Biochem. Biophys. Res. Commun..

[50] Bouskra D (2008). Lymphoid tissue genesis induced by commensals through NOD1 regulates intestinal homeostasis. Nature.

[51] Ramanan D, Tang MS, Bowcutt R, Loke P, Cadwell K (2014). Bacterial sensor Nod2 prevents inflammation of the small intestine by restricting the expansion of the commensal Bacteroides vulgatus. Immunity.

[52] Wang H (2015). New role of nod proteins in regulation of intestinal goblet cell response in the context of innate host defense in an enteric parasite infection. Infect. Immun..

[53] Kim D (2016). Nod2-mediated recognition of the microbiota is critical for mucosal adjuvant activity of cholera toxin. Nat. Med..

[54] Rangan KJ (2016). A secreted bacterial peptidoglycan hydrolase enhances tolerance to enteric pathogens. Science.

[55] Holtje JV (1998). Growth of the stress-bearing and shape-maintaining murein sacculus of Escherichia coli. Microbiol. Mol. Biol. Rev..

[56] Shen B (2014). Efficient genome modification by CRISPR-Cas9 nickase with minimal off-target effects. Nat. Methods.

[57] Stull ND, Breite A, McCarthy R, Tersey SA, Mirmira RG (2012). Mouse islet of Langerhans isolation using a combination of purified collagenase and neutral protease. J. Vis. Exp..

[58] Ai C (2014). Genetically engineered Lactococcus lactis protect against house dust mite allergy in a BALB/c mouse model. PLoS One.

[59] Furet JP, Quenee P, Tailliez P (2004). Molecular quantification of lactic acid bacteria in fermented milk products using real-time quantitative PCR. Int. J. Food Microbiol..

[60] Haarman M, Knol J (2006). Quantitative real-time PCR analysis of fecal Lactobacillus species in infants receiving a prebiotic infant formula. Appl. Environ. Microbiol..

[61] Duttaroy A (2004). Muscarinic stimulation of pancreatic insulin and glucagon release is abolished in m3 muscarinic acetylcholine receptor-deficient mice. Diabetes.

[62] Ediger BN (2017). LIM domain-binding 1 maintains the terminally differentiated state of pancreatic beta cells. J. Clin. Invest..

