# Supplementary material for: Intestinal lysozyme liberates Nod1 ligands from microbes to direct insulin trafficking in pancreatic beta cells
Source: Cell Res. 2019 Jun 14;29(7):516–32. doi: 10.1038/s41422-019-0190-3 (PMC6796897; doi:10.1038/s41422-019-0190-3)
Supplement: Supplementary file 1 — Supplementary information, Figure S1 [file 41422_2019_190_MOESM1_ESM.pdf]

Supplemental Information

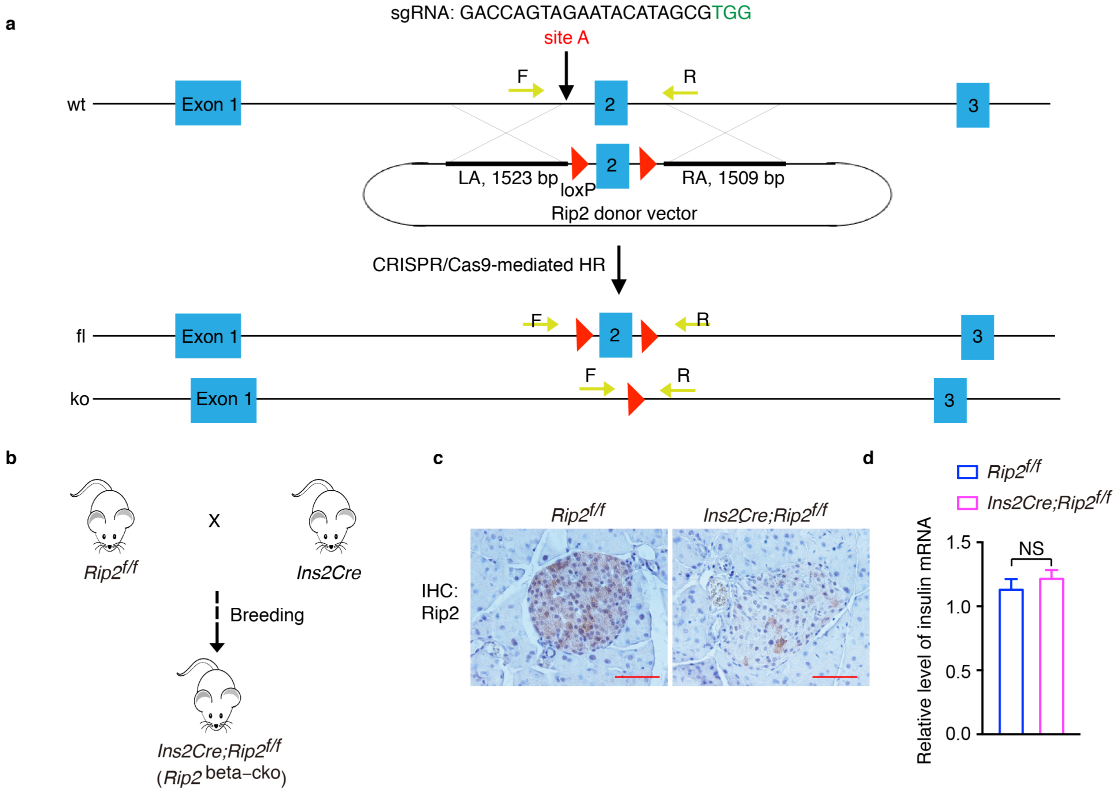

Supplementary information, Fig. S1. Strategy for generating beta cell-specific *Rip2* knockout mice

(a) The targeting strategy for generating the *Rip2* conditional allele with the CRISPR/Cas9 technique. In the donor vector, *loxP* sites are indicated as red triangles. The vector contains ~1500-bp homologous arms on both sides of the loxP-floxed exon (LA, left homology arm; RA, right homology arm). Following Cre-mediated recombination, exon 2 flanked by the two *loxP* sites (red triangles) will be removed. The primers used for genotyping are indicated by yellow arrows. The wild-type, floxed and knockout alleles are depicted.

(b) A schematic of the breeding strategy to produce beta cell-specific *Rip2* knockout mice.

(c) Immunohistochemistry (IHC) analysis of Rip2 in in paraffin sections of pancreases from mice of the indicated genotypes. Scale bars, 50 μm.

(d) Relative levels of insulin mRNA in isolated islets from *Rip2$^{f/f}$* and *Ins2Cre;Rip2$^{f/f}$* mice. PCR primers were designed to measure the total insulin mRNA level, including RNA transcripts from *Ins1* and *Ins2*.

Data (c-d) are representative of two independent experiments. Data in (d) show the mean + s.e.m from one of two independent experiments (n = 3 per group). *P* values were calculated with Student's t test (d) (NS, not significant).
