# Supplementary material for: Intestinal lysozyme liberates Nod1 ligands from microbes to direct insulin trafficking in pancreatic beta cells
Source: Cell Res. 2019 Jun 14;29(7):516–32. doi: 10.1038/s41422-019-0190-3 (PMC6796897; doi:10.1038/s41422-019-0190-3)
Supplement: Supplementary file 2 — Supplementary information, Figure S2 [file 41422_2019_190_MOESM2_ESM.pdf]

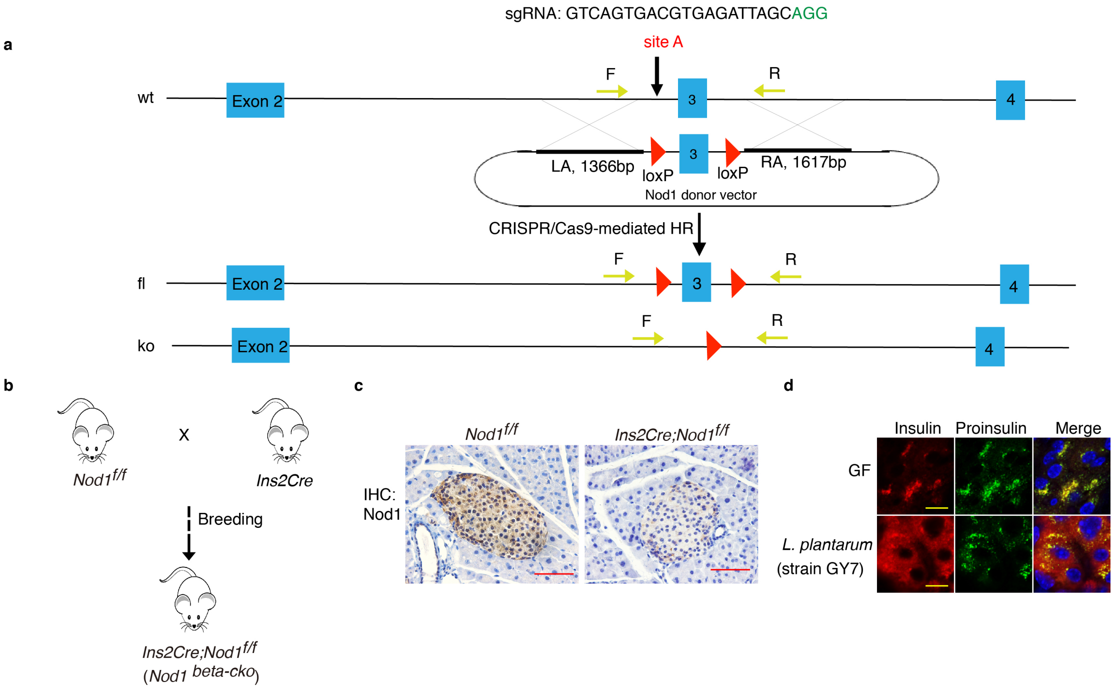

Supplementary information, Fig. S2. Strategy for generating beta-cell specific Nod1 knockout mice

(a) The targeting strategy for generating the *Nod1* conditional allele with the CRISPR/Cas9 technique. In the donor vector, *loxP* sites are indicated as red triangles. The vector contains a ~1300-bp homologous arm on the left side and a ~1600-bp homologous arm on the right side of the loxP-floxed exon (LA, left homology arm; RA, right homology arm). Following Cre-meidated recombination, the exon 3 flanked by two *loxP* sites (red triangles) will be removed. The Primers used for genotyping are indicated by yellow arrows. The wild-type, floxed, and knockout alleles are depicted.

(b) A schematic of breeding strategy to produce beta-cell specific *Nod1* knockout mice.

(c) Immunohistochemistry (IHC) analysis of Nod1 in paraffin sections of pancreases from mice of the indicated genotypes. Scale bars, 50 μm.

(d) Confocal microscopy analysis of insulin (red) and proinsulin (green) in paraffin sections of pancreases from mice mono-colonized with *L. plantarum* strain GY7, or mock-treated (PBS).

Data (c-d) are representative of at least three independent experiments.
