# Supplementary material for: Intestinal lysozyme liberates Nod1 ligands from microbes to direct insulin trafficking in pancreatic beta cells
Source: Cell Res. 2019 Jun 14;29(7):516–32. doi: 10.1038/s41422-019-0190-3 (PMC6796897; doi:10.1038/s41422-019-0190-3)
Supplement: Supplementary file 3 — Supplementary information, Figure S3 [file 41422_2019_190_MOESM3_ESM.pdf]

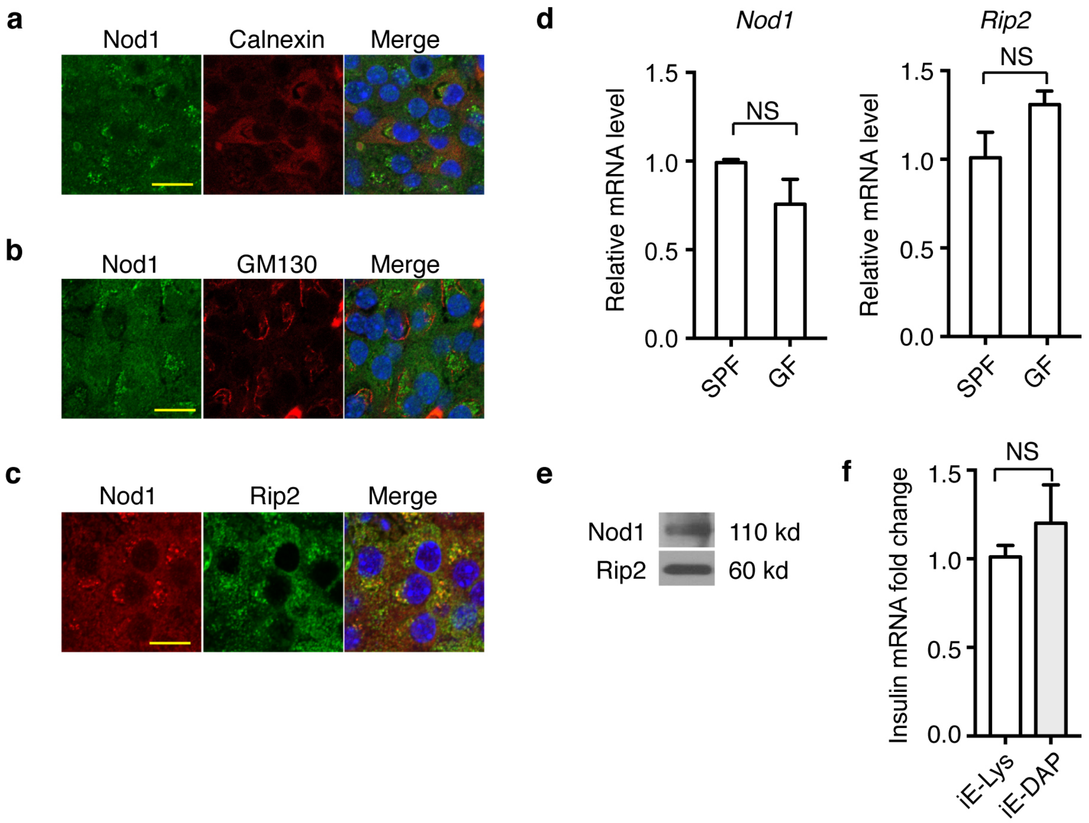

Supplementary information, Fig. S3. The cellular localization of Nod1 and Rip2

(a) Confocal microscopy analysis of Nod1 (green) and calnexin (red) in a paraffin section of pancreas from WT mice.

(b) Confocal microscopy analysis of Nod1 (green) and GM130 (red) in a paraffin section of pancreas from WT mice.

(c) Confocal microscopy analysis of Nod1 (red) and Rip2 (green) in a paraffin section of pancreas from WT mice.

(d) Relative levels of *Nod1* and *Rip2* mRNA in isolated islets from SPF or GF mice.

(e) Immunoblotting of Nod1 and Rip2 in a cell lysate from INS-1 cells.

(f) The relative level of *insulin* mRNA in INS-1 cells treated with iE-DAP or iE-Lys.

Data are representative of at least three independent experiments. Scale bars, 10 μm (a-c). Data in (d and f) show the mean + s.e.m from one of three independent experiments (n = 3-4 per group). *P* values were calculated with Student's t test (d and f) (NS, not significant).
