# Supplementary material for: Intestinal lysozyme liberates Nod1 ligands from microbes to direct insulin trafficking in pancreatic beta cells
Source: Cell Res. 2019 Jun 14;29(7):516–32. doi: 10.1038/s41422-019-0190-3 (PMC6796897; doi:10.1038/s41422-019-0190-3)
Supplement: Supplementary file 4 — Supplementary information, Figure S4 [file 41422_2019_190_MOESM4_ESM.pdf]

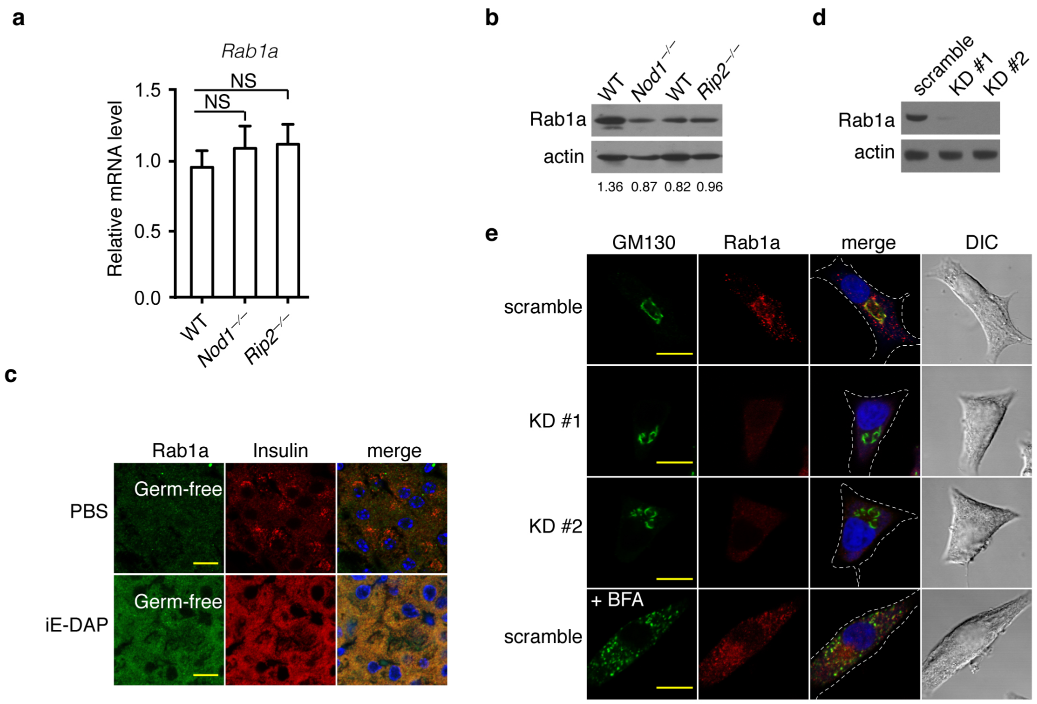

Supplementary information, Fig. S4. Rab1a is recruited to DCVs

(a) The relative level of Rab1a mRNA in isolated islets from WT, *Nod1*$^{-/-}$ or *Rip2*$^{-/-}$ mice.

(b) Immunoblotting of Rab1a in a cell lysate from WT, *Nod1*$^{-/-}$ and *Rip2*$^{-/-}$ islets. Numbers below the lane indicate the relative densitometry between Rab1a and Actin in corresponding lanes.

(c) Confocal microscopy analysis of Rab1a (green) and insulin (red) in paraffin sections of pancreases from GF mice treated with PBS or iE-DAP. Scale bars, 10 μm.

(d) Immunoblotting of Rab1a in cell lysates from a control INS-1 cell line which stably expresses scramble shRNA, or two INS-1 Rab1a knockdown lines (KD #1 and KD #2), which stably express shRNAs against Rab1a. Actin was used as the loading control.

(e) Confocal microscopy analysis of GM130 (green) and Rab1a (red) in INS-1 cells with scramble shRNA, KD #1 or KD #2. Brefeldin A (BFA) treatment was included as a positive control for Golgi fragmentation. The cell boundaries are outlined with white dashed lines. Scale bars, 10 μm.

Data in (a-e) are representative of at least three independent experiments. Data in (a) show the mean + s.e.m from one of three independent experiments (n = 3-4 per group). *P* values were calculated with a one-way ANOVA followed by Tukey's post hoc tests (a) (NS, not significant).
