# Supplementary material for: Intestinal lysozyme liberates Nod1 ligands from microbes to direct insulin trafficking in pancreatic beta cells
Source: Cell Res. 2019 Jun 14;29(7):516–32. doi: 10.1038/s41422-019-0190-3 (PMC6796897; doi:10.1038/s41422-019-0190-3)
Supplement: Supplementary file 5 — Supplementary information, Figure S5 [file 41422_2019_190_MOESM5_ESM.pdf]

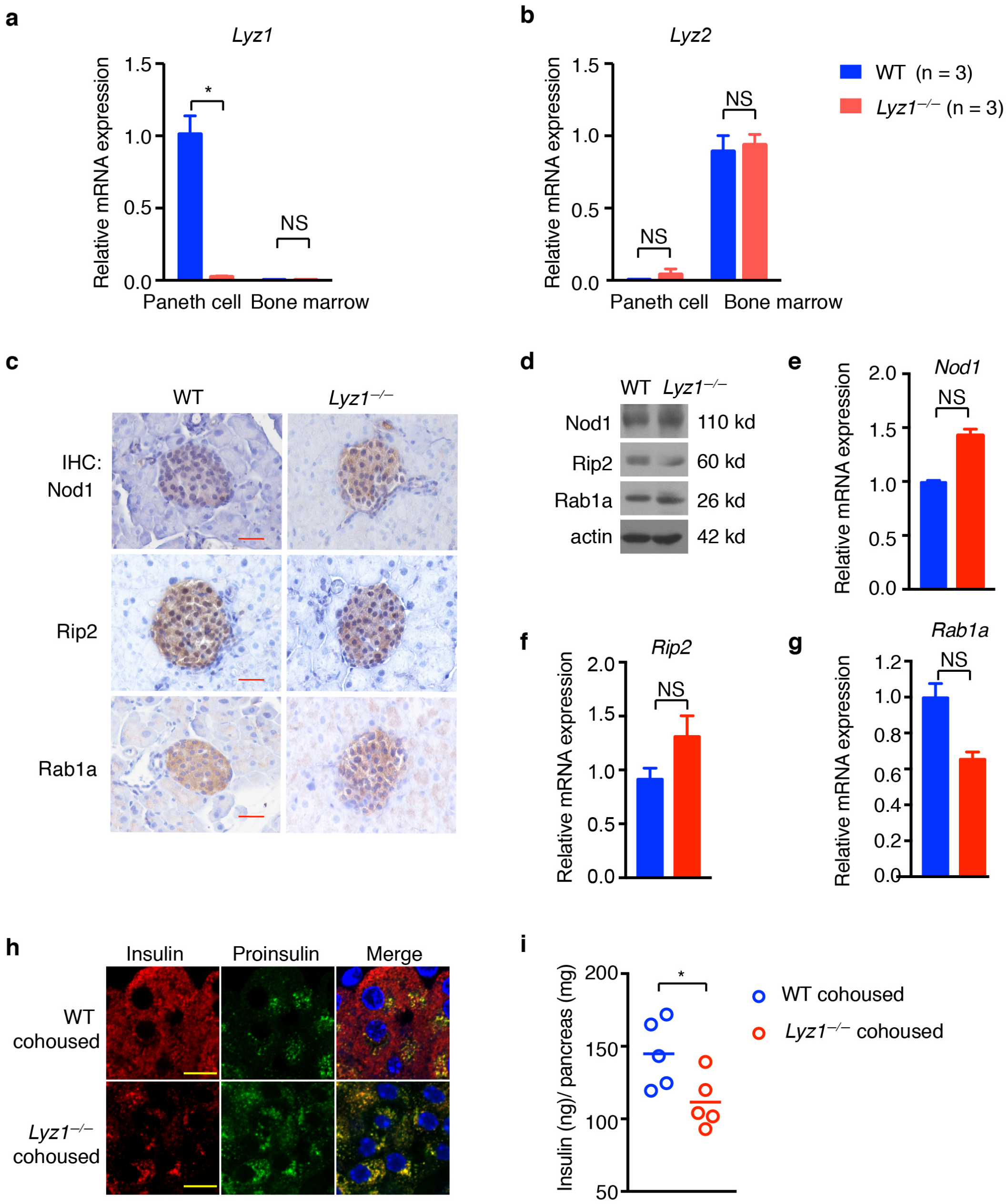

Supplementary information, Fig. S5. Quantification of lysozyme mRNA levels in isolated cells and relative expression of proteins

(a and b) The relative levels of *Lyz1* (a) and *Lyz2* (b) mRNA in Paneth cells and bone marrow cells.

(c) Immunohistochemistry (IHC) analysis of Nod1, Rip2 and Rab1a in paraffin sections of pancreases from mice of the indicated genotypes. Scale bars, 50 μm.

(d) Immunoblotting analysis of Nod1, Rip2 and Rab1a in isolated islets from WT or *Lyz1*$^{-/-}$ mice. Actin was used as loading control.

(e-g) The relative levels of *Nod1* (e), *Rip2* (f) and *Rab1a* (g) mRNA in isolated islets from WT or *Lyz1*$^{-/-}$ mice.

(h) Confocal microscopy analysis of insulin (red) and proinsulin (green) in paraffin sections of pancreases from co-housed WT and *Lyz1*$^{-/-}$ mice. Scale bars, 10 μm.

(i) The insulin content in pancreatic tissues from co-housed WT and *Lyz1*$^{-/-}$ littermates.

Data are expressed as the mean of three individual animals + s.e.m (a, b, e-g). Each symbol indicates each animal, with bars indicating mean. *P* values were calculated with a Student's t-test (a, b, e-g, i), NS, not significant, * $P < 0.05$. Experiments were repeated for three times.
