# Supplementary material for: Intestinal lysozyme liberates Nod1 ligands from microbes to direct insulin trafficking in pancreatic beta cells
Source: Cell Res. 2019 Jun 14;29(7):516–32. doi: 10.1038/s41422-019-0190-3 (PMC6796897; doi:10.1038/s41422-019-0190-3)
Supplement: Supplementary file 6 — Supplementary information, Figure S6 [file 41422_2019_190_MOESM6_ESM.pdf]

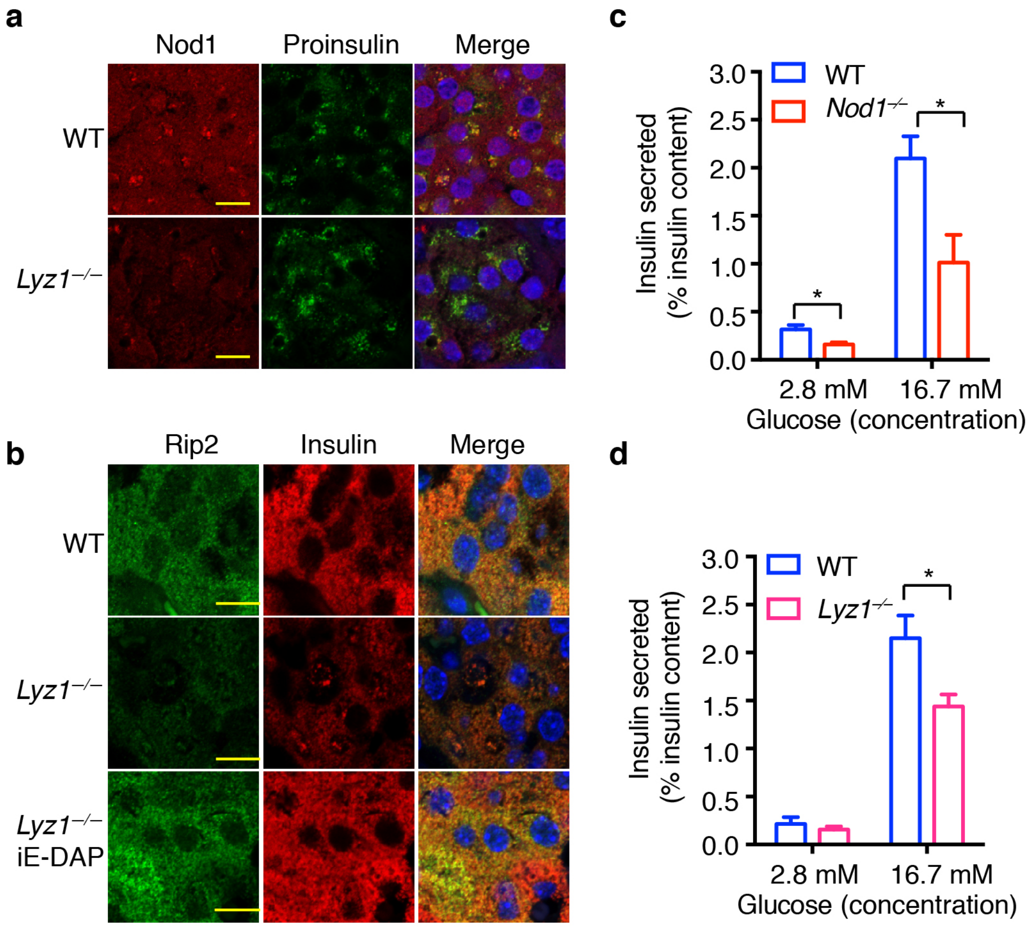

Supplementary information, Fig. S6. The cellular localization of Nod1 and Rip2

(a) Confocal microscopy analysis of Nod1 (red) and proinsulin (green) in pancreatic islet sections from WT and *Lyz1*$^{-/-}$ mice.

(b) Confocal microscopy analysis of Rip2 (green) and insulin (red) in pancreatic islet sections from mice of WT, *Lyz1*$^{-/-}$ and *Lyz1*$^{-/-}$ supplemented with iE-DAP.

(c and d) The amount of insulin secreted from indicated mice islets in an *ex vivo* glucose stimulated insulin secretion (GSIS) assay with batch incubation.

Scale bars, 10 µm. *P* values were calculated with a two-tailed Student's t test, *, $P < 0.05$. Data (a-d) are representative of at least three independent experiments.
