# Supplementary material for: Intestinal lysozyme liberates Nod1 ligands from microbes to direct insulin trafficking in pancreatic beta cells
Source: Cell Res. 2019 Jun 14;29(7):516–32. doi: 10.1038/s41422-019-0190-3 (PMC6796897; doi:10.1038/s41422-019-0190-3)
Supplement: Supplementary file 7 — Supplementary information, Figure S7 [file 41422_2019_190_MOESM7_ESM.pdf]

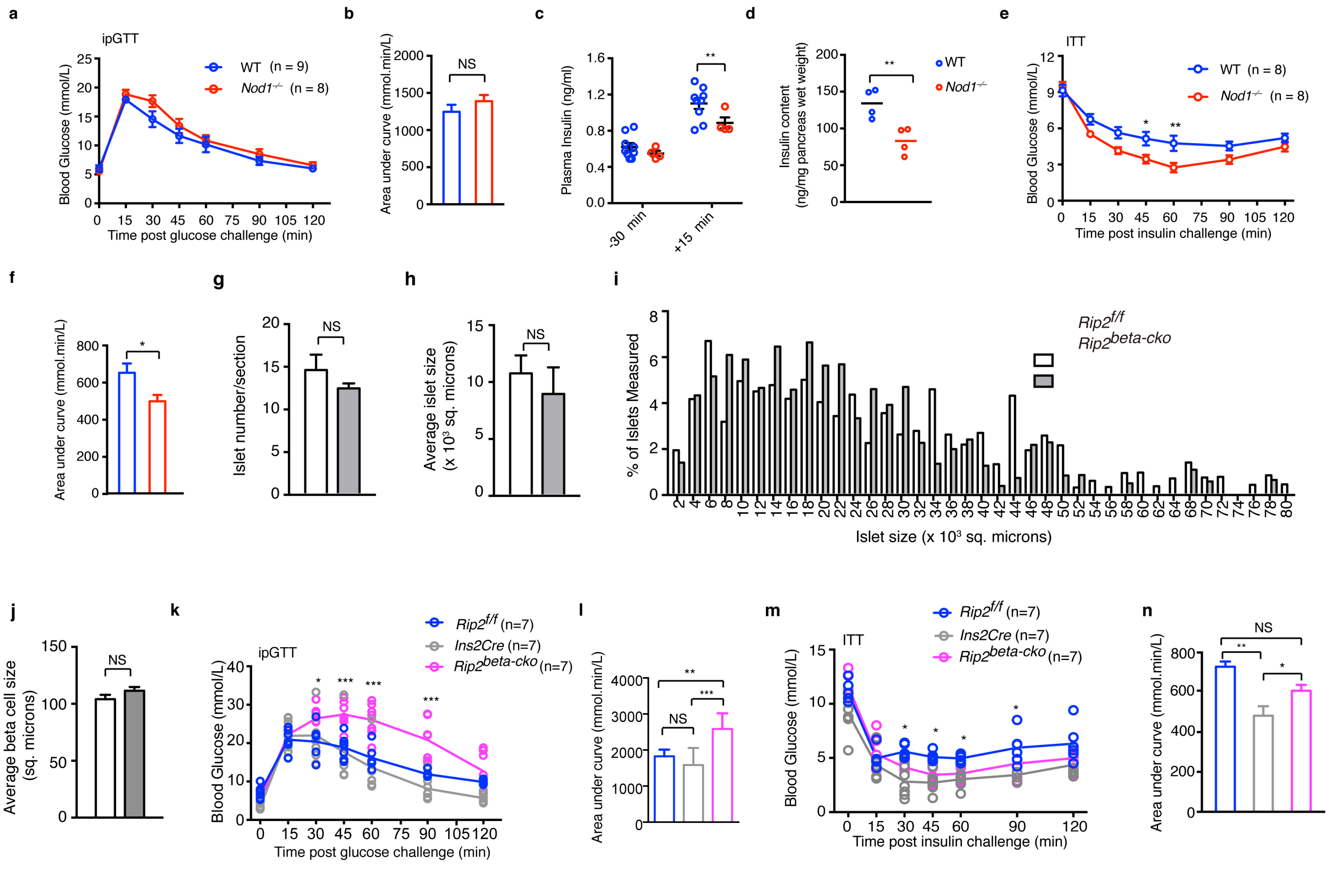
a
ipGTT
WT (n = 9)
Nod1-/- (n = 8)
Blood Glucose (mmol/L)
Time post glucose challenge (min)
b
NS
Area under curve (mmol.min/L)
c
Plasma Insulin (ng/ml)
**
-30 min
+15 min
d
Insulin content (ng/mg pancreas wet weight)
**
WT
Nod1-/-
e
ITT
WT (n = 8)
Nod1-/- (n = 8)
Blood Glucose (mmol/L)
Time post insulin challenge (min)
f
Area under curve (mmol.min/L)
g
Islet number/section
NS
h
Average islet size (x 10³ sq. microns)
NS
i
% of Islets Measured
Rip2f/f
Rip2beta-cko
Islet size (x 10³ sq. microns)
j
Average beta cell size (sq. microns)
NS
k
ipGTT
Rip2f/f (n=7)
Ins2Cre (n=7)
Rip2beta-cko (n=7)
Blood Glucose (mmol/L)
Time post glucose challenge (min)
l
Area under curve (mmol.min/L)
NS
m
ITT
Rip2f/f (n=7)
Ins2Cre (n=7)
Rip2beta-cko (n=7)
Blood Glucose (mmol/L)
Time post insulin challenge (min)
n
Area under curve (mmol.min/L)
NS

Supplementary information, Fig. S7. Nod1 deficiency does not change glucose tolerance but increases relative insulin sensitivity

(a) Concentration of blood glucose during an intraperitoneal GTT (ipGTT) in the indicated mice.

(b) Blood glucose AUC during the ipGTT in (a).

(c) Concentration of plasma insulin in WT and *Nod1*$^{-/-}$ mice 30 minutes before and 15 minutes after glucose challenge.

(d) The amount of insulin in pancreatic tissues from WT and *Nod1*$^{-/-}$ mice.

(e) Concentration of blood glucose during an intraperitoneal ITT (ipITT) with 0.35 IU/kg insulin in the indicated mice.

(f) Blood glucose AUC during the ipITT in (e).

(g) The mean number of islets per section of pancreas from *Rip2*$^{f/f}$ and *Rip2*$^{beta\text{-}cko}$ animals. 2 animals of each genotype were analyzed.

(h) Average islet size in pancreas from *Rip2*$^{f/f}$ and *Rip2*$^{beta\text{-}cko}$ animals. 2 animals of each genotype were analyzed.

(i) Size distribution of pancreatic islets in *Rip2*$^{f/f}$ and *Rip2*$^{beta\text{-}cko}$ animals; the percentage of the total number of islets counted is plotted against islet size. 2 animals of each genotype were analyzed.

(j) The average beta cell size (sq. microns) in *Rip2*$^{f/f}$ and *Rip2*$^{beta\text{-}cko}$ animals. 2 animals of each genotype were analyzed.

(k) Concentration of blood glucose during an intraperitoneal GTT in the indicated mice.

(l) Blood glucose AUC during the ipGTT in (k).

(m) Concentration of blood glucose during an intraperitoneal ITT in the indicated mice.

(n) Blood glucose AUC during the ipITT in (m).

Each symbol represents mean of individual animals in a group, and bars indicate s.e.m (a, b, e, f, l, n). Each symbol represents an individual animal, and horizontal bars indicate median values (c, d, k, m). *P* values were calculated with a two-way ANOVA followed by Tukey's post hoc tests (a, e, k, m), a one-way ANOVA followed by Tukey's post hoc tests (c, l, n) and a Student's T test (b, f, g, h, j). * $P < 0.05$; ** $P < 0.01$, *** $P < 0.001$; NS, not significant. Data are representative of three independent experiments (a-j).
