# Supplementary material for: Intestinal lysozyme liberates Nod1 ligands from microbes to direct insulin trafficking in pancreatic beta cells
Source: Cell Res. 2019 Jun 14;29(7):516–32. doi: 10.1038/s41422-019-0190-3 (PMC6796897; doi:10.1038/s41422-019-0190-3)
Supplement: Supplementary file 8 — Supplementary information, Figure S8 [file 41422_2019_190_MOESM8_ESM.pdf]

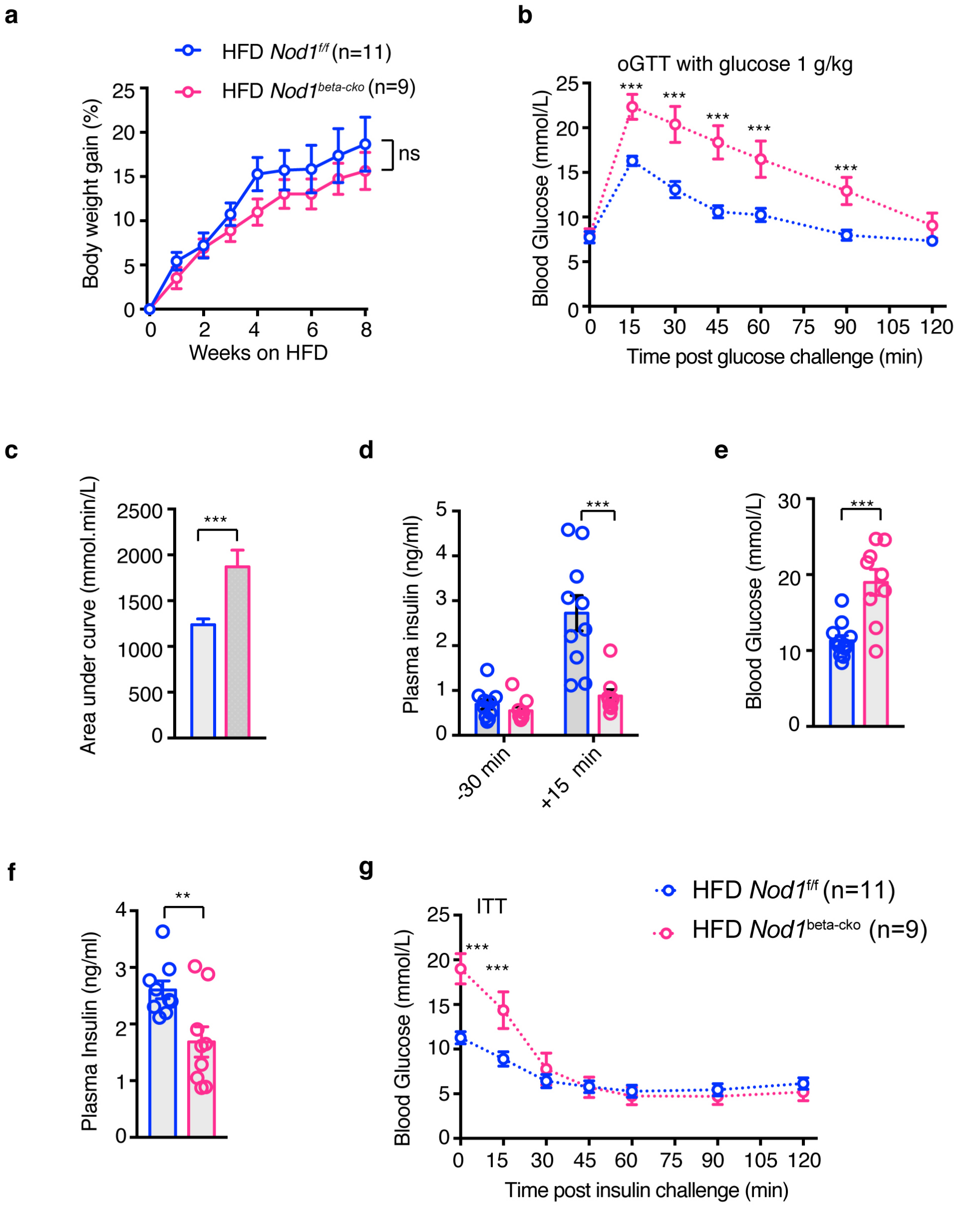

Supplementary information, Fig. S8. The lack of the Nod1 ligand-mediated crosstalk can further aggravate hyperglycemia associated with HFD

(a) Body weight changes of *Nod1*$^{f/f}$ and *Nod1*$^{beta\text{-}cko}$ mice during 8 weeks of HFD.

(b, c) Concentration of blood glucose during an oGTT in the indicated mice (b) and the area under the curve from (c). To avoid blood sugar levels exceeding the measurement range of the GlUCOCARD blood glucose meter, 1 g/kg glucose was orally administrated in HFD-fed animals.

(d) Concentration of plasma insulin in the indicated mice 30 minutes before and 15 minutes after oGTT.

(e, f) Blood glucose level (e) and plasma insulin level (f) in 6-hour-starved *Nod1*$^{f/f}$ and *Nod1*$^{beta\text{-}cko}$ mice after 8 weeks of HFD.

(g) Concentration of blood glucose during an intraperitoneal ITT in *Nod1*$^{f/f}$ and *Nod1*$^{beta\text{-}cko}$ mice after 8 weeks of HFD.

Each symbol represents mean of individual animals in a group, and bars indicate sem (a, b, g). Each symbol represents an individual animal, and lines or horizontal bars indicate median values (d-f). Data are representative of two independent experiments (a-g). *P* values were calculated with a two-way ANOVA followed by Tukey's post hoc tests (a, b, g), one-way ANOVA followed by Tukey's post hoc tests (d), a two-tailed Student's t test (c, e, f). (ns, not significant; ** $P < 0.01$; *** $P < 0.001$).
