# Supplementary material for: Intestinal lysozyme liberates Nod1 ligands from microbes to direct insulin trafficking in pancreatic beta cells
Source: Cell Res. 2019 Jun 14;29(7):516–32. doi: 10.1038/s41422-019-0190-3 (PMC6796897; doi:10.1038/s41422-019-0190-3)
Supplement: Supplementary file 9 — Supplementary information, Table S1 [file 41422_2019_190_MOESM9_ESM.pdf]

Supplementary information, Table S1. Nucleotide Sequence

**qPCR primers**	
Insulin-F	TGGCTTCTTCTACACACCCAAG
Insulin-R	ACAATGCCACGCTTCTGCC
Lyz1-F	CCCAGGCCAAGGTCTACAAT
Lyz1-R	ATCCCACAGGCATTCTTAGA
Lyz2-F	CTCAGGCCAAGGTCTATGAA
Lyz2-R	ATCCCACAGGCATTCACAGC
Nod1-F	GGTCACCAACATTCGGAACA
Nod1-R	CTTTCGGACCTTGTCAGGC
Rip2-F	ATCCCGTACCACAAGCTCG
Rip2-R	GGATGTGTAGGTGCTTCACTG
Rab1a-F	CAACAGCAAAGGAATTTGCAG
Rab1a-R	GCTTGACTGGAGTGCTCTGGA
Gapdh-F	TGTTCCTACCCCCAATGTGT
Gapdh-R	GGTCCTCAGTGTAGCCCAAG
**Rab1a knockdown oligos**	
Rat Rab1a shRNA scramble F	GATCCCCGCGCGCTTTGTAGGATTCGTTCAA GAGACGAATCCTACAAAGCGCGCC TTTTTA
Rat Rab1a shRNA scramble R	AGCTTAAAAAGCGCGCTTTGTAGGATTCGTC TCTTGAACGAATCCTACAAAGCGCGCCGGG
Rat Rab1a shRNA 1# F	GATCCCCGGTTGGAAAGTCTTGCCTTCTTTCAA GAGAAGAAGGCAAGACTTTCCAACCTTTTTA
Rat Rab1a shRNA 1# R	AGCTTAAAAAGGTTGGAAAGTCTTGCCTTCTTC TCTTGAAAGAAGGCAAGACTTTCCAACCGGG
Rat Rab1a shRNA 2# F	GATCCCCGGATGACACGTATACGGAAAGTTCA GAGACTTTCCGTATACGTGTCATCC TTTTTA
Rat Rab1a shRNA 2# R	AGCTTAAAAAGGATGACACGTATACGGAAAGC TCTTGAACTTTCCGTATACGTGTCATCCGGG
**Bacterial specific primers**	
Lactococcus lactis-F	GCAATTGCATCACTCAAAGA
Lactococcus lactis-R	ACAGAGAACTTATAGCTCCC
Lactobacillus plantarum-F	TGGATCACCTCCTTTCTAAGGAAT
Lactobacillus plantarum -R	TGTTCTCGGTTTCATTATGAAAAAATA
**Primers for *Lyz1* knockout mice**	
Lyz1 sgRNA oligo-F	TAGGGGTCTACAATCGTTGTGAGT
Lyz1 sgRNA oligo-R	AAACACTCACAACGATTGTAGACC
Lyz1 ko genotyping-F	AGAACGTAATACGAAGACATG
Lyz1 ko genotyping-R	TCTGAGCCATCTCTCCAGCC
**Potential off-target site sequencing primers**	
Spaca3 seq-F	TCTCCACCCTTTCACTTCT
Spaca3 seq-R	GGCTGTCAATCTTCCCTC

Sec14L2 seq-F	TTGCTGAGCCTCTTTGTC
Sec14L2 seq-R	CTGTCTGAGCTTCCGTGT
Gria1 seq-F	AGACAGGGACCCAGTTGC
Gria1 seq-R	AGGAGTGAGGTGGAAAGC
**Primers for *Nod1* knockout mice**	
Nod1 sgRNA oligo-F	TAGGAACATCTGGTCACCAACATT
Nod1 sgRNA oligo-R	AAACAATGTTGGTGACCAGATGTT
Nod1 ko genotyping-F	GATGGAGAATTCACCAGGGA
Nod1 ko genotyping-R	AAATAGCCCAGAGACCACAG
**Potential off-target site sequencing primers**	
Kctd12b seq-F	GATCAGATGGGTCATTAGGC
Kctd12b seq-R	CATTTCCAATTGAGTTGAGC
Abhd16b seq-F	GATGTAGTGGTCAAATATGCG
Abhd16b seq-R	GCTAAGCACCACTCATCATC
Mcf2 seq-F	CTGAGCAACTGCCAGAAAGT
Mcf2 seq-R	TTCTCCAGGAGACATTTCAC
Etnk1 seq-F	CAGTGCATAAGCACGTGAAG
Etnk1 seq-R	GCTCCCAAAGCCAATTTCAT
Sbsn seq-F	GAAGGAGACACAGAGAGTTG
Sbsn seq-R	GGGTTTTCTCAGCCACTATC
**Targeting sequence and primers for *Rip2*$^{f/f}$ mice**	
Targeting sequence	GACCAGTAGAATACATAGCGTGG
genotyping-F	AAACAGACCAGTAGAATA
genotyping-R	GAAGTAAAAGAAATTCAC
**Targeting sequence and primers for *Nod1*$^{f/f}$ mice**	
Targeting sequence	GTCAGTGACGTGAGATTAGCAGG
genotyping-F	CTCCTGTTACCCTCCTGC
genotyping-R	AGACAGAGTCGCTCCACA
