# Supplementary material for: Intestinal lysozyme liberates Nod1 ligands from microbes to direct insulin trafficking in pancreatic beta cells
Source: Cell Res. 2019 Jun 14;29(7):516–32. doi: 10.1038/s41422-019-0190-3 (PMC6796897; doi:10.1038/s41422-019-0190-3)
Supplement: Supplementary file 10 — Supplementary information, Table S2 [file 41422_2019_190_MOESM10_ESM.pdf]

Supplementary information, Table S2. Key Resources Table

REAGENTS OR RESOURCES	SOURCE	IDENTIFIER
**Antibodies**		
Anti-HA (Co-IP)	Sigma-Aldrich	Cat# H3663
Anti-Myc (9E10) (Co-IP)	Santa Cruz Biotechnology	Cat# sc-40
Anti-Insulin (IF, IHC)	Abcam	Cat# ab7842
Anti-Proinsulin (IF)	HyTest	Cat# 2PR8-CCI-17
Anti-Nod1 (WB)	Zen BioScience	Cat# 501246
Anti-Nod1 (IF, IHC)	Novus Biologicals	Cat# NB100-56878
Anti-Nod2 (IF, IHC)	Santa Cruz Biotechnology	Cat# sc-30199
Anti-Rip2 (6F7) (WB)	Abnova Corporation	Cat# H00008767-M02
Anti-Rip2 (IF, IHC)	Abcam	Cat# ab8427
Anti-Rab1a (WB, IF, IHC)	Proteintech Group	Cat# 11671-1-AP
Anti-GM130 (IF)	BD Biosciences	Cat# 610822
Anti-Calnexin (IF)	BD Biosciences	Cat# 610523
Anti-Lysozyme (IHC)	Zen BioScience	Cat# 617544
Goat anti-Guinea pig IgG - H&L Polyclonal Secondary Antibody, Biotin	Abcam	Cat# ab6907
Goat anti-Guinea Pig IgG (H+L) secondary antibody, TRITC	Thermo Fisher	Cat# A18774
Goat anti-Rabbit IgG (H+L) Cross-Adsorbed Secondary Antibody, Alexa Fluor 555	Thermo Fisher	Cat# A21428
Goat anti-Mouse IgG (H+L) Secondary Antibody, Alexa Fluor 555	Thermo Fisher	Cat# A21422
Goat anti-Rabbit IgG (H+L) Secondary Antibody, Alexa Fluor 488	Thermo Fisher	Cat# A11008
Goat anti-Mouse IgG (H+L) Cross-Adsorbed Secondary Antibody, Alexa Fluor 488	Thermo Fisher	Cat# A11001
Goat anti-Rat IgG (H+L) Cross-Adsorbed Secondary Antibody, Alexa Fluor 488	Thermo Fisher	Cat# A11006
Goat anti-Mouse IgG (H+L) pAb-HRP	MBL	Cat# 330
Goat anti-Mouse Kappa-HRP	Southern Biotech	Cat# 1050-05
Goat anti-Mouse IgG1, Human ads-HRP	Southern Biotech	Cat# 1070-05
Mouse anti-Rabbit IgG-HRP	Southern Biotech	Cat# 4090-05

**Reagents**		
Normal goat serum blocking reagent	Boster Biotech	Cat# AR1009
DAPI	Novon	Cat# SS0156
Fluoromount-G	Southern Biotech	Cat# 0100-01
DAB Peroxidase (HRP) substrate	Maixin Biotech	Cat# DAB-0031
Hemotoxylin solution	Sigma-Aldrich	Cat# 03971
DYNA beads protein G	Thermo Fisher	Cat# 10004D
NUPAGE 10% Bis-Tris Gel	Thermo Fisher	Cat# NP0302BOX
PageRuler Prestained protein Ladder	Thermo Fisher	Cat# 26616
Pure nitrocellulose blotting membrane	PALL	Cat# P/N 66485
Luminata Forte Western HRP substrate	Millipore	Cat# WBLUF0100
DMEM	Hyclone	Cat# sh3002201B
FBS	Bioind	Cat# 04-001-1A
RPMI 1640 Medium	Gibco	Cat# 11875-093
Endotoxin free PBS	Gibco	Cat# 10010-023
Glutamine	Gibico	Cat# 25030-081
HEPES	Gibico	Cat# 15630-080
Accutase	Gibico	Cat# A11105-01
Collagenase XI	Sigma-Aldrich	Cat# C7657
Hexadimethrine bromide (polybrene)	Sigma-Aldrich	Cat# H9268
Lipofectamine 2000	Thermo Fisher	Cat# 11668019
Trizol	Thermo Fisher	Cat# 15596018
Brefeldin A	Medchemexpress	Cat# HY-16592
Fatty acid free BSA	Sigma-Aldrich	Cat# A8806
Muramic acid	Sigma-Aldrich	Cat# M2503
LPS	Sigma-Aldrich	Cat# L2630
iE-Lys	InvivoGen	Cat#tlrl-lys
iE-DAP	InvivoGen	Cat# tlrl-dap
Tri-DAP	InvivoGen	Cat# tlrl-tdap
MDP	Sigma-Aldrich	Cat# A9519-25MG
PGN (*Bacillus subtilis*)	Sigma-Aldrich	Cat# 69554-10MG-F
Lysozyme human recombinant	Sigma-Aldrich	Cat# L1667-1G
Insulin human recombinant	Sigma-Aldrich	Cat# I3536
**Reagent kits**		

PrimeScript RT reagent Kit	TAKARA	Cat# DRR047A
SYRB Premix Ex Taq	TAKARA	Cat# DRR820A
MEGAshortscript T7 kit	Thermo Fisher	Cat# AM1354
T7 Ultra Kit	Thermo Fisher	Cat# AM1345
MEGAclear kit	Thermo Fisher	Cat# AM1908
Insulin ELISA kit	Millipore	Cat# EZRMI-13K
Proinsulin ELISA kit	Alpco	Cat# 80-PINMS-E01
UltraSensitive TM S-P Kit mouse	Maixin Biotech	Cat# KIT-9701
UltraSensitive TM S-P Kit rabbit	Maixin Biotech	Cat# KIT-9706
Dual Glo Luciferase Reporter Assay system	Promega	Cat# E2920
**Recombinant DNA**		
pST1374-Cas9 vector	Addgene	Cat# 44758
PUC57-sgRNA vector	Addgene	Cat# 51132
pSuper.Retro.Puro vector	OligoEngine	Cat# VEC-PRT-0002
pcDNA3.1(+) vector	Thermo Fisher	Cat# V790-20
pCMV-myc vector	Clontech	Cat# 631604
Rip2 cDNA cloned from mouse crypts	This study	N/A
Rab1a cDNA cloned from mouse lung	This study	N/A
**Experimental Models: Cell Lines**		
Rat: INS-1	China Infrastructure of Cell Line Resources	Cat# 3111C0001CCC000378
Human: HEK293T	ATCC	Cat# CRL-3216
Human: HEK/mNOD1 cells	InvivoGen	Cat# hkb-mnod1
Human: 293/mNOD2 cells	InvivoGen	Cat# 293-mnod2
**Experimental Models: Organisms/strain**		
Mouse: *Rip2*$^{-/-}$	Jackson laboratory	Cat# 007017
Mouse: *Myd88*$^{-/-}$	Jackson laboratory	Cat# 009088
Mouse: *Nod1*$^{-/-}$	This study	N/A
Mouse: *Nod2*$^{-/-}$	RIKEN	Cat# 01869
Mouse: *Lyz1*$^{-/-}$	This study	N/A
Mouse: *Ins2Cre*	Jackson laboratory	Cat# 003573
Mouse: *Rip2*$^{f/f}$	This study<br>Casgene Biotech Company	N/A
Mouse: *Nod1*$^{f/f}$	This study	N/A

	Casgene Biotech Company	
Lactobacillus plantarum (strain NY)	Gift from Jin Zhong (Institute of Microbiology, CAS)	N/A
Lactobacillus plantarum (strain GY7)	Gift from Jin Zhong (Institute of Microbiology, CAS)	N/A
Lactococcus lactis (strain NZ9000)	Gift from Wei Chen (Jiang Nan University)	N/A
**Software and Algorithms**		
GraphPad Prism v7.0a	GraphPad software	https://www.graphpad.com/scientificsoftware/prism
Image J v1.48	NIH	https://imagej.nih.gov/ij/
Zeiss Zen Imaging software (blue edition)	Zeiss	https://www.zeiss.com/microscopy/int/software-cameras.html
**Sequence-based reagents**		
Primers, see **Table S1**	This study	
